# Dissecting the cell to nucleus, perinucleus and cytosol

**DOI:** 10.1038/srep04923

**Published:** 2014-05-12

**Authors:** Tattym E. Shaiken, Antone R. Opekun

**Affiliations:** 1 Department of Molecular and Cellular Oncology, University of Texas M. D. Anderson Cancer Center, Houston, TX 77030, USA; 2 Departments of Medicine & Pediatrics G.I. & S.A.H.S. Baylor College of Medicine-McNair Faculty Center A10.019 One Baylor Plaza (GI Medicine MS901), Houston, Texas 77030, USA

## Abstract

Cells have been described under the microscope as organelles containing cytoplasm and the nucleus. However, an unnoted structure exists between the cytoplasm and the nucleoplasm of eukaryotic cells. In addition to the nuclear envelope, there exists a perinuclear region (PNR or perinucleus) with unknown composition and function. Until now, an investigation of the role of the perinucleus has been restricted by the absence of a PNR isolation method. This manuscript describes a perinucleus isolation technique on the basis of its unique compact organization. The perinucleus was found to contain approximately 15 to 18% of the total proteins of the mammalian cell, almost half of the proteins of nuclei. Using four different normal and cancer cell lines, it was shown that the composition of PNR is highly dynamic. Application of the method showed that translocation of the p53 tumor-suppressor protein to the perinucleus in immortalized MEF cells is correlated with the translocation of p53-stabilizing protein, nucleophosmin (B23), to the PNR. Herein, the concept of the perinuclear region is advanced as a formal, identifiable structure. The roles of the perinucleus in maintaining genome integrity, regulation of gene expression and understanding of malignant transformation are discussed.

The detection of the perinuclear space within the bi-layered nuclear membrane of the cell was accomplished by microscopic imagining long ago^1^. Bundles of filaments were observed encircling the nucleus, but were not well described. In cultured endothelial cells obtained from the guinea pig, the filaments were measured by electron microscopy to be 100 Å in diameter with unknown function^2^. Studies that examined the perinuclear region of cells showed that perinuclear translocation of certain proteins and enzymes was essential for their proper functioning and depended upon growth factors^3^ or other stimuli^4^. It was determined that STAT3 localized sequentially to endocytic vesicles, in the cytosol or at the perinuclear region following PDGF treatment^3^. It was shown that upon prolonged stimulation by an agonist, NT1 receptors transiently accumulated in the perinuclear recycling compartment using the microtubule network^4^. Accumulation of BCL10 at the perinuclear region was shown to be required for BCL10-mediated NF-κB activation^5^. Hu and Exton (2004) demonstrated that only the unphosphorylated form of PKCα can colocalize and activate PLD1 at the perinuclear region following PMA stimulation^6^ and that phenylalanine 663, in the C-terminus, was required for perinuclear translocation^7^. Reinecke J.B. et al (2014) demonstrated that inactive proto-oncogene tyrosine-protein kinase Src (Src) is localized in the perinuclear endocytic recycling compartment (ERC), but growth factor stimulation promotes the release of Src from the ERC and translocates Src protein to the plasma membrane, where it triggers downstream cellular processes^8^.

However, the significance of the perinuclear region was not well appreciated for its signal modulation role until now. The functioning of Src and other kinases, linked to cancer progression, might be dependent upon perinuclear dynamics. Although it is known that the nuclear envelope is the borderline of nuclei, it remains unclear what protects genome integrity from the damaging signals originating from receptors when signals go awry. In addition to the nuclear envelope, between the cytoplasm and nucleoplasm, there exists a perinuclear region with unknown structure and function. A thorough investigation of the role of the perinuclear region has been restricted by the absence of proper isolation techniques because the borders of the perinucleus are not delimited by a membrane structure. Here we describe isolation of the perinucleus of a cell using a chemical fractionation method and discuss its role in genome integrity and in the transmission of cytoplasmic signals to the nucleus.

## Results

### The essence of the cellular dissection (CDS) technique

**Figure 1:**
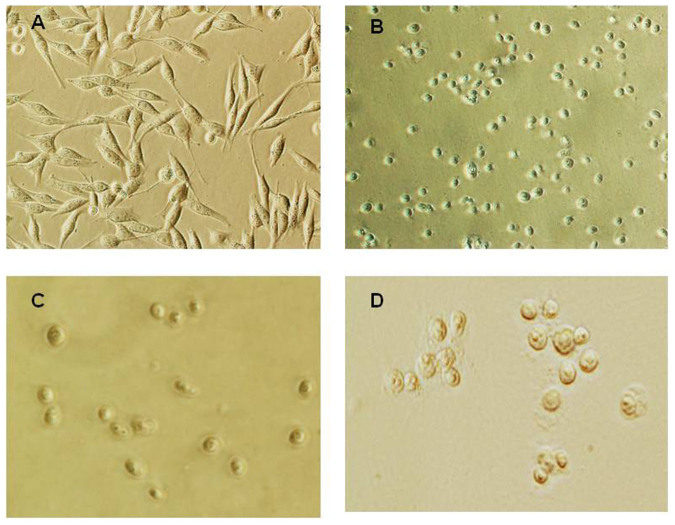
Phase contrast images of MDA-MB-435 cells and isolated nuclei. (A) MDA-MB-435 cells. (B) Nuclei isolated in isotonic buffer A. Cytoplasm of the cell at this stage was removed by detergent-containing buffer; approximately 65% of proteins were extracted to the cytosol; nuclei contain perinuclear region proteins; nucleoli are visible. (C) Nuclei after extraction of the perinuclear region with buffer B. The core nucleus does not collapse after removal of the perinuclear region proteins; approximately 20% of total cellular proteins were extracted with the perinuclear fractionation; nucleoli are visible. (D) Nuclei isolated with the classical method of using hypotonic buffer. The shape of nuclei varies; some “fibrous” structures around nuclei are visible; nuclei contain nucleoli.

In order to isolate the perinuclear region, the melanoma-derived MDA-MB-435 cell line was used as a model^9^ (Figure 1A). MDA-MB-435 cells were lysed with Buffer A and the nuclei were sequentially washed with low- and no-detergent containing buffer A (Figure 1B). The perinuclear proteins were extracted with buffer B (Figure 1C) and the core nuclear fraction (cNF) was dissolved in 8 M urea. The nuclei also were isolated with the classical scheme of fractionation in hypotonic buffer^10^, which was used as a control (Figure 1D). As a control for the fractionation, Chaps-containing buffer^11^ was applied for cell lysis. Buffer A resulted in extraction of approximately 70% of the cytoplasmic protein of the cell (Table 1). Further fractionation of nuclei (shown in Figure 1B) extracted approximately 15–18% more proteins, which are believed to compose the compact perinuclear (PNF) fraction (Table 1). Cancer cell lines MDA-MB-435 and HeLa and immortalized MEF cells contained higher concentrations of protein in the PNF than primary MEF cells. After PNF extraction, it was observed that the nuclei did not collapse and retained the nucleoli (Figure 1C), accounting for approximately 15% of the total cellular protein (Table 1). Total nuclear proteins of the perinuclear fraction and core nuclear fractions (PNF + cNF) compose approximately 30% of total cellular proteins.

**Figure 2:**
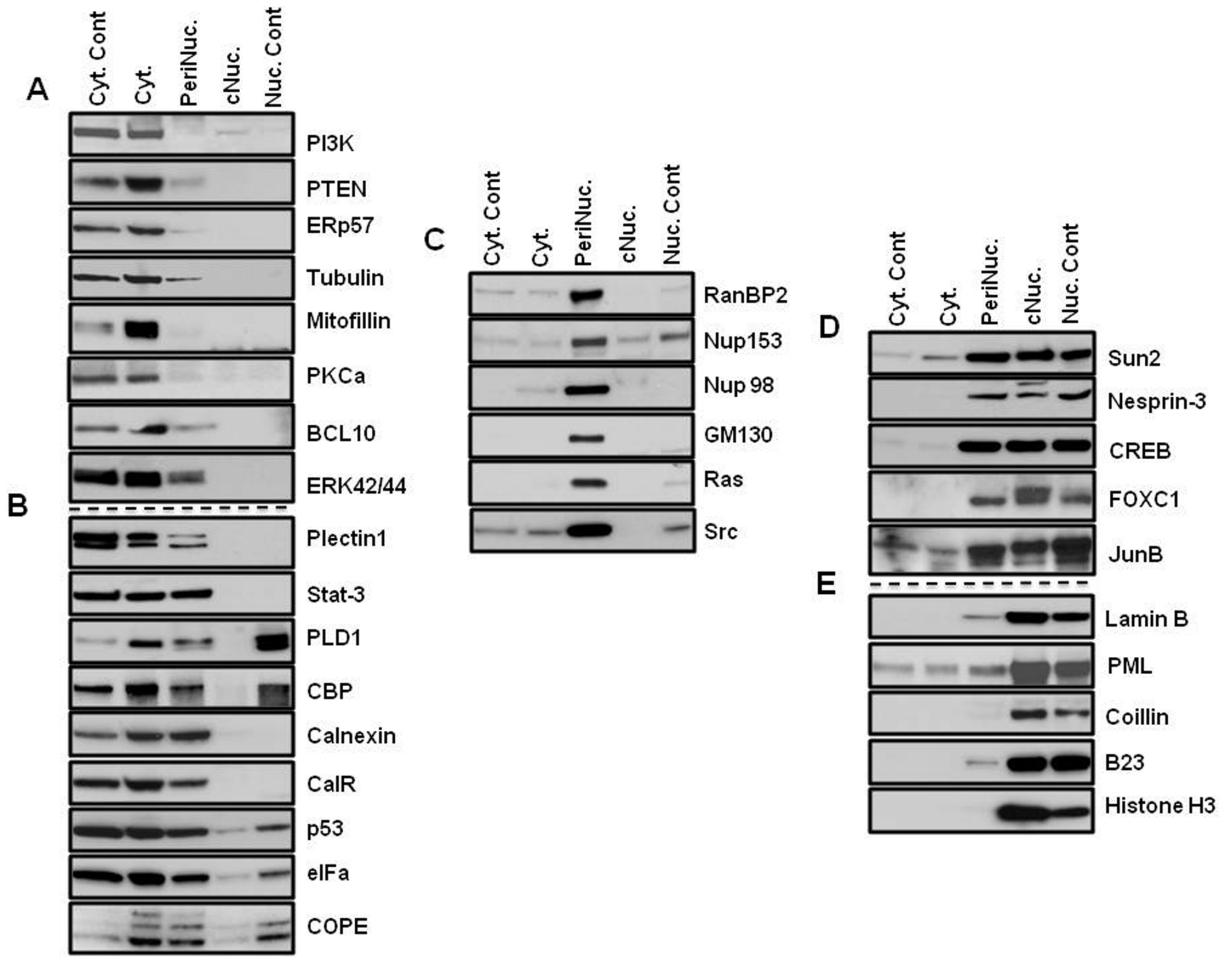
Patterns of protein distribution in MDA-MB-435 cells. Cyt. Cont is a control for cytosolic proteins obtained with the 0.3% Chaps buffer cell lysis (far left panel of bands) for CDS method; Nuc. Cont is a control for nuclear proteins obtained with classical method of nuclei isolation in hypotonic buffer (far right panel of bands) for CDS method. (A) Proteins of cytosol: proteins extracted by regular lysis buffer and Buffer A from cytoplasm. They are not detected in perinuclear and nuclear fractions. Nuclear fractions were obtained with new and classical nuclei extraction techniques (B) Proteins detected in the cytosol and the perinuclear fraction: proteins were detected as cytosolic proteins with both cellular lysis technique; in addition, these proteins also appeared in the perinuclear fraction by extraction with Buffer B. (C) Proteins of perinuclear fraction: proteins are detected only in the perinuclear fraction by buffer B extraction. (D) Proteins detected in nuclear and perinuclear fractions: transcription factors (that supposedly belong to the nuclear fraction) also appeared in the perinuclear fraction. (E) Nuclear proteins: proteins were detected in the nuclear fraction. Nuclear proteins were obtained with both new and classical nuclei isolation techniques. The PVDF membranes were cropped into two halves and the high and low molecular weight proteins were shown correspondingly.

**Figure 3:**
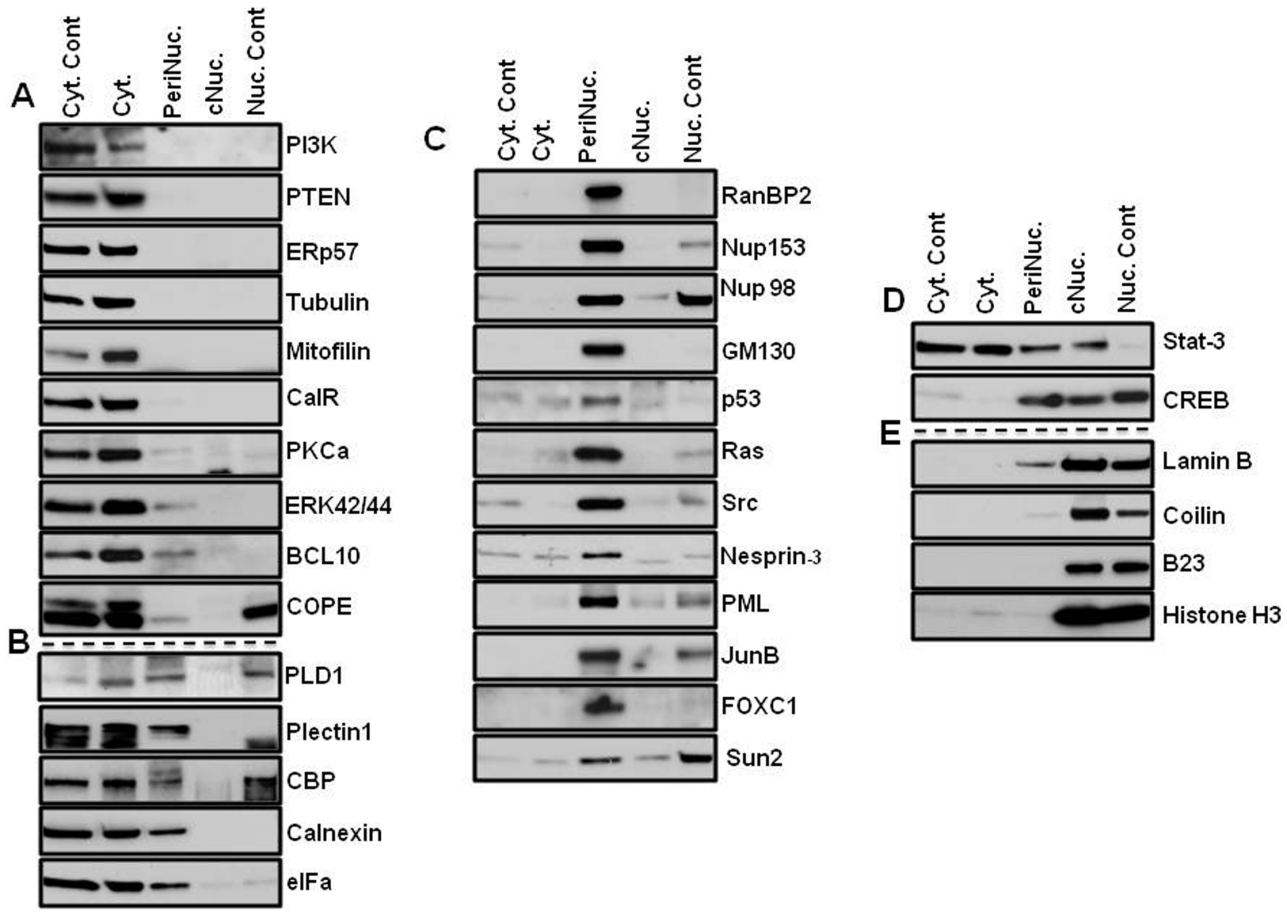
Patterns of protein distribution in HeLa cells. Cyt. Cont is a control for cytosolic proteins obtained with the 0.3% Chaps buffer cell lysis (far left panel of bands) for the CDS method; Nuc. Cont is a control for nuclear proteins obtained with the classical method of nuclei isolation in hypotonic buffer (far right panel of bands) for the CDS method. (A) Proteins of cytosol: proteins extracted by regular lysis buffer and Buffer A from the cytoplasm. They are not detected in the perinuclear and the nuclear fractions. Nuclear fractions were obtained with new and classical nuclei extraction techniques (B) Proteins detected in the cytosol and the perinuclear fraction: proteins were detected as cytosolic proteins with both cellular lysis technique; in addition, these proteins also appeared in the perinuclear fraction by extraction with Buffer B. (C) Proteins of perinuclear fraction: proteins are detected only in perinuclear fraction by buffer B extraction. p53 protein was detected with long exposure. (D) Proteins detected in the nuclear and the perinuclear fractions: transcription factor CREB appeared in both fractions. (E) Nuclear proteins: proteins were detected in nuclear fraction. Nuclear proteins were obtained with new and classical nuclei isolation techniques. The PVDF membranes were cropped into two halves and the high and low molecular weight proteins were shown correspondingly.

**Figure 4:**
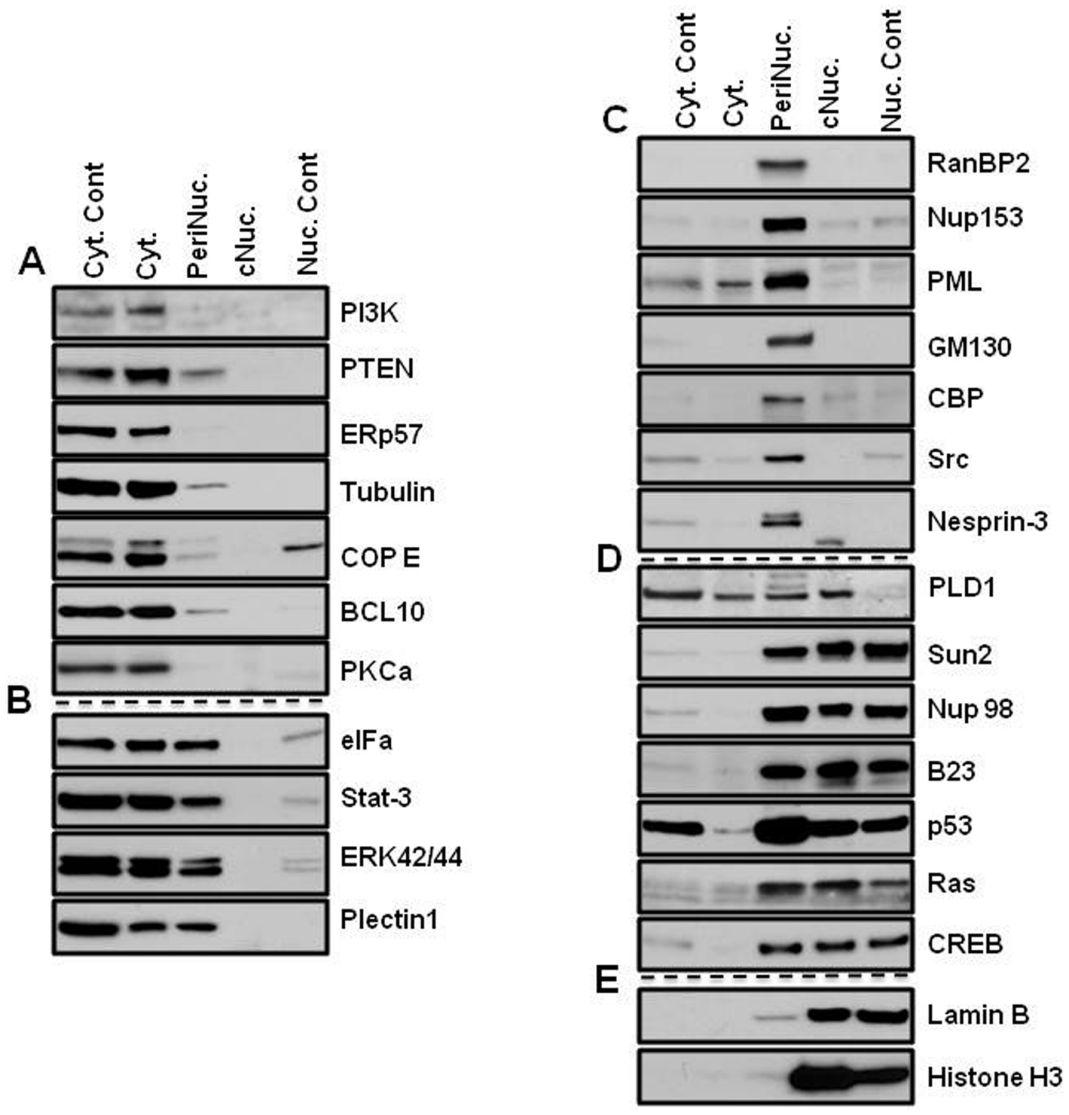
Patterns of protein distribution in MEF cells. Cyt. Cont is a control for cytosolic proteins obtained with the 0.3% Chaps buffer cell lysis (far left panel of bands) for the CDS method; Nuc. Cont is a control for nuclear proteins obtained with classical method of nuclei isolation in hypotonic buffer (far right panel of bands) for the CDS method. (A) Proteins of cytosol: proteins extracted by regular lysis buffer and Buffer A from the cytoplasm. They are not detected in perinuclear and nuclear fractions. Nuclear fractions were obtained with new and classical nuclei extraction techniques. (B) Proteins detected in the cytosol and the perinuclear fraction: proteins were detected as cytosolic proteins with both cellular lysis technique; in addition, these proteins also appeared in the perinuclear fraction by extraction with Buffer B. (C) Proteins of the perinuclear fraction: proteins are detected only in the perinuclear fraction by buffer B extraction. CBP protein was detected with long exposure. (D) Proteins detected in nuclear and perinuclear fractions: transcription factor CREB appeared in both fractions, in addition Ras and p53 proteins were detected. (E) Nuclear proteins: Nuclear proteins were obtained with new and classical nuclei isolation techniques. The PVDF membranes were cropped into two halves and the high and low molecular weight proteins were shown correspondingly.

**Figure 5:**
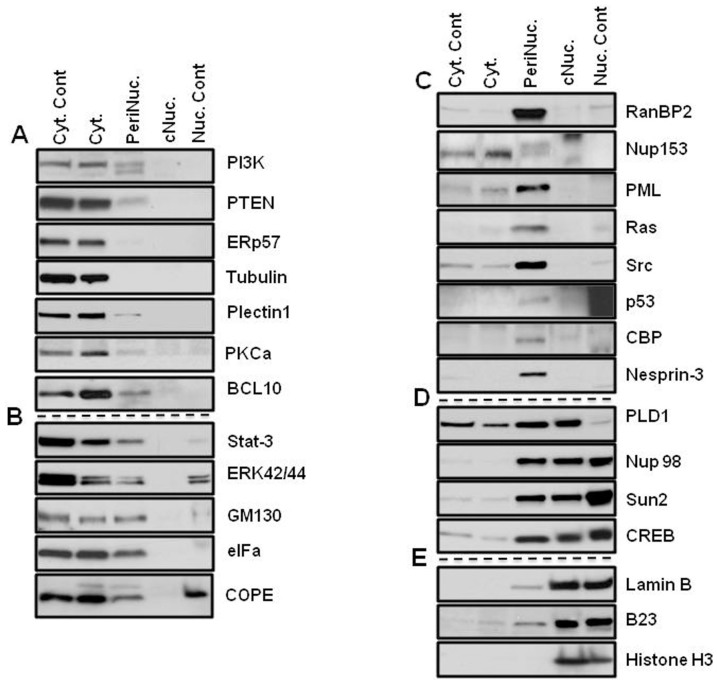
Patterns of protein distribution in Primary MEF cells. Cyt. Cont is a control for cytosolic proteins obtained with the 0.3% Chaps buffer cell lysis (far left panel of bands) for the CDS method; Nuc. Cont is a control for nuclear proteins obtained with the classical method of nuclei isolation in hypotonic buffer (far right panel of bands) for the CDS method. (A) Proteins of cytosol: proteins extracted by regular lysis buffer and Buffer A from the cytoplasm. They are not detected in the perinuclear and the nuclear fractions. Nuclear fractions were obtained with new and classical nuclei extraction techniques. (B) Proteins detected in the cytosol and the perinuclear fractions: proteins were detected as cytosolic proteins with both cellular lysis technique; in addition, these proteins also appeared in the perinuclear fraction by extraction with Buffer B. (C) Proteins of the perinuclear fraction: proteins are detected only in the perinuclear fraction by buffer B extraction. Ras, p53 and CBP proteins were detected with long exposure. (D) Proteins detected in the nuclear and the perinuclear fractions: transcription factor CREB appeared in both the nuclear and the the perinuclear fractions. (E) Nuclear proteins: proteins were detected in nuclear fraction. Nuclear proteins were obtained with new and classical nuclei isolation techniques. The PVDF membranes were cropped into two halves and the high and low molecular weight proteins were shown correspondingly.

A slight difference was visible under the phase contrast microscope between nuclei fractionated with the different nuclear-extraction techniques (Figure 1C, D). While nuclei that were isolated with the classical technique showed some fibrous structures around the nuclei, the PNF-extracted nuclei did not. To address the question of how much the perinuclear fraction was contaminated with cytosolic or nuclear content, the marker proteins of the fractions were analyzed by immunoblotting. In order to distinguish the perinuclear fraction from other cellular compartments, cells were lysed, and the nuclei were isolated as described above, after which the PNF was extracted and analyzed (Figures 2,3,4,5). The PNF and other cellular fractions from two cancer cell lines and two mouse embryonic fibroblasts (immortalized and primary fibroblasts) were tested (Table 2).

### Subdivision of the cellular proteins

Proteins detected by immunoblot analysis were arbitrarily divided into five groups depending on their cellular localization:

- Group (A): soluble proteins of cytosol, plasma membrane proteins, and proteins of membranous structures of cytoplasm;
- Group (B): proteins, which are detected in both cytosol and in the perinucleus;
- Group (C): proteins localized only in the perinucleus;
- Group (D): proteins that may shuttle between the perinucleus and the nucleus; and
- Group (E): nuclear proteins;

A PI3K p110 alpha isoform^12^ was detected only in the cytosolic fractions obtained with both lysing techniques, which included Chaps and NP-40 detergents in all cell lines (Figures 2A,3A,4A,5A). There was an exception for the primary mouse embryonic fibroblasts (pMEF), in which the protein appeared as a double band in the PNF, although at much lower concentrations. The same pattern of localization revealed the antagonistic partner of the PI3K pathway, PTEN^13^; however, a much smaller amount of this phosphatase also was detected in the perinuclear region of MDA-MB-435 and MEF cells.

ERp57, an endoplasmic reticulum-resident protein^14^, as well as mitofilin (a mitochondrial transmembrane protein)^15^, were extracted from the cytosolic fraction in MDA-MB-435 and HeLa cells (Figures 2A and 3A), and in MEF cells, ERp57 was detected in the same fraction (Figures 4A and 5A). Mitofilin was not detected in MEF cells since the antibody does not react with mouse protein (*a list of antibodies that do not cross-react with mouse proteins is shown in the material section*). The cytoskeletal protein α−Tubulin^16^ was detected predominantly in the cytosol fraction (Figures 2A,3A,4A,5A). All of the aforementioned proteins that were not detected in the nuclear fraction were isolated by two different methods: a classical and a cellular-dissection technique.

From the panel of investigated proteins, calreticulin^17^, a multifunctional protein that binds the Ca^2+^ ion, was detected only in the cytosolic fraction in HeLa cells (Figure 3A). In MDA-MB-435 cells, calreticulin was present in the perinuclear and in the cytosolic fraction (Figure 2B). The difference in calreticulin distribution between the HeLa and MDA-MB-435 cells is probably a result of the tissue specificity of the investigated cell lines^18^.

COPE showed predominantly cytosolic localization in HeLa and MEF cells (Figures 3A, 4A); however, the most significant detection was in the perinuclear fraction in MDA-MB-435 cells and in primary MEF cells (Figures 2B and 5B). COPE coatomer is a cytosolic protein complex that reversibly associates with Golgi non-clathrin-coated vesicles. It is required for budding from Golgi membranes^19^ and is essential for retrograde Golgi-to-ER transport. Although COPE was not present in the nucleus after fractionation by the cellular-dissection method, it was well detected in the nucleus by the classical nuclear-isolation method. The main discrepancy between these two methods is iso- versus hypotonic conditions of nuclei isolation. Expansion of the nuclear size during osmotic shock may cause partial disruption of the nuclear envelope, which might allow mixing of the cytoplasmic and nuclear contents of cells. Similar patterns of nuclear distribution differences were found for p53 and eIFα in MDA-MB-435 cells (Figure 2B) and for CBP in HeLa cells (Figure 3B).

Another group of proteins showed close distribution in the cytosol and the perinuclear fractions: CBP^20^, a CREB-binding protein, was abundant in the cytosol and in the perinuclear fractions of MDA-MB-435 and HeLa cells (Figures 2B and 3B). However, in MEF cells, CBP was observed only in the perinuclear fraction (Figures 4C and 5C). In primary MEF cells, this protein was barely detected at long exposure. CPB is a coactivator of CREB^21^, and its slow migratory form was detected in the PNF of HeLa cells.

Calnexin^22^, calreticulin, p53^23^ tumor suppressor, and eIFα, an initiating factor, were equally abundant in the perinuclear fraction as in the cytosolic fraction in MDA-MB-435 cells (Figure 2B). Calnexin was also abundant in the PNF of HeLa cells (Figure 3B), whereas eIFα demonstrated high concentration in the perinuclear region of all types of investigated cells (Figures 2B,3B,4B,5B).

Plectin is a large protein (>500 kDa) that acts as a link between the three main components of the cytoskeleton: actin microfilaments, microtubules (MT) and intermediate filaments (IFs). Plectin 1 an Plectin isoform is equipped with a C-terminal high-affinity IF-binding site that can mediate the targeting and anchorage of IFs at clearly defined cellular locations^24^. Plectin 1 was found predominantly in cytosolic fraction of primary MEF cells (Figure 5A); however, in three other investigated cells (Figures 2B,3B,4B), it was also clearly detected in the perinuclear fraction. Nesprin-3, one of six KASH proteins, binds to plectin, which in turn links to the actin and/or intermediate filaments^25^. In this study, Nesprin-3 was detected only in the perinuclear fraction of HeLa and MEF cells (Figure 3C,4C,5C) and predominantly in the PNF and to a lesser degree in nuclear fractions of MDA-MB-435 cells (Figure 2D). SUN proteins are type II membrane proteins that have previously been described as being exclusively localized in the inner nuclear membrane (INM)^26^. In this study, SUN-2 protein was almost equally distributed between the nuclear and perinuclear fractions of MDA-MB-435 and MEF cells (Figures 2D, 4D, and 5D); slightly more protein was extracted from the PNF of HeLa cells (Figure 3C) during CDS fractionation.

Nuclear pore complex proteins RanBP2/Nup358^27^, Nup153^28^, and a marker of the Golgi complex, GM130^29^, were extracted only in the perinuclear fraction in all of the studied cell types (Figures 2C,3C,4C), with the exception of the GM130 of primary mouse fibroblasts, where it was also detected in the cytosol (Figure 5B). The Golgi complex is probably an integral part of the perinuclear region in cancerous and immortalized cell lines. Nup153 also was extracted in the cytosolic fraction of primary cells; however, its slow migratory band stayed in the PNF. Another nuclear pore complex protein, Nup98, nucleoporin, that is localized to both the nuclear and the cytoplasmic sides of the NPC^30^, was found in only the PNF of MDA-MB-435 and HeLa cells (Figures 2C and 3C) and in both the PNF and the nuclear fractions of both MEF cells (Figures 4D and 5D). Nuclear laminas interact with membrane-associated proteins to form the nuclear lamina on the interior of the nuclear envelope. B-type lamins are anchored to the inner nuclear membrane via a prenylated cysteine residue at the C-terminus^31^. Lamin B was found only in the nuclear fraction of all four investigated cells (Figures 2,3,4,5E).

Signal transducers and activators of transcription 3, also known as STAT3, are phosphorylated by receptor-associated kinases in response to cytokines and growth factors and then translocate to the cell nucleus, where they act as transcription activators^32^. STAT3 was detected predominantly in cytosolic fraction of primary MEF cells, although a less amount is present in the PNF (Figure 5B). STAT3 was equally distributed in the cytosolic and perinuclear fractions of MDA-MB-435 and MEF cells (Figures 2B and 4 B) and was almost equally distributed in the cytosolic, perinuclear and nuclear fractions of HeLa cells (Figure 3D).

In HeLa cells, transcription factors JunB^33^ and FOXC1^34^ were restricted in the perinuclear fraction; however, in MDA-MB-435 cells, these proteins were abundant in the nuclear fraction as well (Figures 2D and 3C). Nuclear FOXC1 in MDA-MB-435 also had a slow migratory component, but the perinuclear protein shows only one fast migratory band. Because the MDA-MB-435 cells originated from melanoma, FOXC1 and JunB might be more functionally active in MDA-MB-435 cells than in HeLa cells. B-cell lymphoma/leukemia 10 (BCL10) has been shown to contain a caspase recruitment domain (CARD), which has been shown to induce apoptosis and to activate NF-kB^35^. BCL10 has been detected as a cytosolic protein, although a lesser amount of protein was observed in the perinuclear fraction (Figures 2A,3A,4A,5A).

Phospholipase D (PLD) has been shown be regulated by many factors, including protein kinase C (PKC) and small G-proteins of the Rho and ADP-ribosylation factor families^36^. In this study, PLD1 was detected in all cellular fractions of MEF cells; in primary cells it was more concentrated in the PNF (Figure 5D), but in immortalized MEF cells, slow migratory bands were observed in the PNF (Figure 4D). In MDA-MB-435 and HeLa cells, it also appeared in the cytosole (Figues 2B and 3B).

PKCα, a cytoplasmic protein and member of protein kinase C family, has been shown to translocate to the perinuclear region following PMA stimulation^6^. In this study, and under non-stimulated conditions, PKCα was only detected in the cytosolic fraction of all investigated cell lines (Figures 2A,3A,4A,5A).

Promyelocytic leukemia protein^37^ was limited in the perinuclear fraction in HeLa and MEF cells (Figures 3C,4C,5C), although in MDA-MB-435 cells, it was abundant in the nucleus (Figure 2E). Immunoblot detection revealed that Histone H3^38^, B23^39^ and coilin^40^ are nuclear proteins (Figures 2E,3E,4E,5E). Protein p44/42 MAPK or ERK p42/44, are ubiquitous in the cellular-signaling cascades^41^. ERK p42/44 was detected predominantly in the cytosol of MDA-MB-435 and HeLa cells (Figures 2A and 3A), but in MEF cells showed cytosolic and perinuclear localization (Figures 4B, and 5B).

Ras^42^ oncoprotein was found in the perinuclear fraction of all investigated cells (Figures 2C, 4C, and 5C); however, in immortalized MEF cells, it was also present in the nuclear fraction (Figure 4D). Src is a non-receptor protein tyrosine kinase that phosphorylates specific tyrosine residues in other proteins, and elevated c-Src activity has been linked to cancer progression^43^. In this study, Src protein was detected predominantly in the perinuclear fraction of all cells (Figure 2C,3C,4C,5C).

Transcription factor p53 was only detected in the perinuclear and nuclear fractions in MEF cells (Figure 4D), whereas CREB^21^ transcription factor was detected in the perinuclear and nuclear fractions of all cells (Figures 2D,3D,4D,5D). Comparison of the presence of suppressor p53 and Ras oncoproteins showed startling differences in immortalized MEF cells and in primary mouse embryonic fibroblasts. These proteins were abundant in the nucleus and the perinucleus of the transformed MEF cells (Figure 4D); however, in primary MEF cells, they were barely detected in the perinuclear fraction at a long exposure of film (Figure 5C). Suppressor protein p53 was only detected in a long exposure of film in HeLa cells (Figure 3C). The mosaic of specific protein distribution could be a fingerprint of the perinuclear complex of the cell lines and may reflect the difference of the signaling pathways in different cell lines.

## Discussion

### The rationale for perinucleus isolation

In order to study the cellular structure that protects and regulates the dynamics and integrity of the cellular genome, a technique that allows extraction of the perinuclear region and isolation of the nucleus was developed.

A classical nuclear-isolation technique exploits the ability of hypotonic solution to swell cells such that the expanded plasma membranes cannot maintain their integrity, allowing leakage of the cytoplasmic contents. However, the resilience of the nuclear membrane retains its nuclear content^10^. Application of the technique allows for isolation of pure nuclei, but some steps must be performed with optimal timing to prevent rupture of the nuclear membrane^44^. In these experiments, even with optimal timing, minor mixing of the nuclear/cytosolic components occurred. As an example, COPE, eIFα, and CBP were detected in the nuclear fraction isolated with the classical nuclei isolation technique, whereas CDS isolation did not.

The classical method of nuclear isolation relies on the concept that the nucleus is surrounded with a highly organized structure that is able to tolerate the expanding force of moderate molecular diffusion. It is difficult to appreciate how a solitary bi-layer lipid structure might hold up against the osmotic pressure of hypotonic buffer unless the perinuclear region provides additional support to the nuclear envelope with a highly proteinaceous fabric. The composition of this barrier appears to involve both the nuclear envelope and the perinuclear region of the nucleus comprises approximately half of total nuclear proteins. A thorough review of the literature did not adequately reveal descriptions of the perinuclear structure and function.

In order to isolate the perinuclear region, the classical technique was reexamined and cells were treated with isotonic buffer instead of hypotonic buffer. The method permitted extraction of the perinuclear fractions without destruction of the core nuclei, and the nucleoplasm retained the nucleoli, nuclear bodies and transcriptional factors.

### The mosaic of the perinuclear proteins may reflect cell differentiation status

After removing the nuclear and cytosolic milieu, the perinuclear fraction showed the presence of diverse functional proteins; however, the level of the subcellular distribution was broad and dependent upon the cell line (Table 2). In the perinuclear fraction, the cellular membrane (PI3K p110-α and PTEN), cytoskeleton (α-Tubulin), endoplasmic reticulum (ERp57), mitochondrial (mitofilin) and nuclear proteins (Lamin B, coilin, and Histone H3) were not detected or were detected at very low concentrations in comparison to the cytosolic fraction.

The technique also permitted extraction of nuclear pore complex proteins. As an indicator of this, RanBP2 and Nup98 were extracted from the cytoplasmic side and Nup153 and Nup98 were extracted from the nuclear side. The resistance of the PNF to Buffer A and an ability of Buffer B to extract the PNF supported the compositional differences of the cytoplasmic and the PNF fractions.

It was concluded that the core nucleus (nucleoplasm) is surrounded not only by the nuclear envelope, but also by a compact, organized structure that includes the nuclear envelope, the nuclear pore complex and various proteins composing approximately 20% of total cellular proteins. The protein proportion of PNF varies depending upon cell type, and the mosaic of specific protein distribution could be a fingerprint of the perinuclear complex. The existence of transcriptional factors in the perinuclear region suggests that the perinuclear complex may be the site for their restriction and also may regulate their nuclear functions.

Investigation of the perinuclear fraction, as a genome-protecting entity, prompts reconsideration of the concepts of the signaling pathways, which descend from the cellular surface to the nucleus. CBP was recently shown to function as cytoplasmic E4 polyubiquitin ligase^20^, and in this study it was found in the perinucleus (Figures 2B, 3B, 4C and 5C). CREB was detected in both the perinuclear and the nuclear fractions in all cell types of normal and cancer cell lines.

The method of PNF and nuclei isolation described herein uncovers a new approach in understanding the signal transmission from the cytoplasm to the nucleus and shows that it may not be necessary for CBP to enter the nucleus for CREB activation. CREB may shuttle between these two fractions, whereas CBP is located in the PNF and locally regulates its activity. The differences in perinuclear composition among different cell types indicate tissue specificity of the cells' origin and differentiation. A number of PNF proteins such as FOXC1, CBP, PI3K 110α, and Nup153 are distinguished by posttranslational modification when their nuclear or cytoplasmic variants are compared. Therefore, the mosaic of perinuclear proteins can be a fingerprint of the cell type and function to protect its genome integrity. It has been demonstrated that translocation of cytosolic proteins, such as PKC^6^, BCL10^5^ and STAT-3^3^, from cytoplasm to the perinucleus are under the effect of growth factors and other stimuli. This shows that perinuclear localization of specific proteins is dynamic and depends upon the growth factor stimulation or cellular stress.

### Perinuclear signal transmission in normal and malignant transformation

A surprising difference in perinuclear localization of p53 and Ras and Src proteins in primary and immortalized cells, as well as in cancer cell lines, underscores an important role of these proteins and provides for a better understanding of the mechanisms of cellular transformation. Tumor suppressor p53 is barely detected in pMEF since it is not up-regulated in a normal cell. However, in viral-transformed MEF cells, p53 was abundant in the PNF and in the nucleus (detected by both nuclei-isolation methods), but was not found in the cytoplasm by the CDS method. Kurki, S. *et al.* (2004) demonstated that nucleophosmin (B23) is an abundant nucleolar phosphoprotein that interacts with tumor suppressor protein p53^45^. Overexpression of p53 and its perinuclear concentration in immortalized MEF correlates with the translocation of B23 into the perinucleus, supporting the dynamic character of the perinucleus and enhancing understanding of the nucleophosmin-mediated stabilization model previously proposed. This work suggests that stabilization of p53 may actually take place in the perinucleus.

In the MDA-MB-435 cancer cell line, mutated^46^ p53 is localized in the PNF and cytosol, not in the nucleus, which suggests that its role as a tumor suppressor is limited. Moreover, in the HeLa cancer cell line, p53 is barely detected and only in the PNF since HPV (human papillomavirus) can interact with p53, resulting in the rapid degradation of p53^47^. Therefore, our method demonstrates that p53 tumor suppressor effect appears dependent upon the PNF localization. None of the investigated cells were Ras transformed. In normal pMEF cells, Ras protein was barely detected in the PNF, but other cell lines showed higher expression of the protein and perinuclear localization, except the immortalized MEF, which contained it in the nucleus, detected with both nuclei-isolation techniques. It is well known that healthy people have cells with oncogenic K-Ras in different organs at rates far exceeding the rates of cancer development, and Magliano and Lonsdon (2013) report that the presence of oncogenic KRAS is not sufficient to transform cells, but the mechanism that removes barriers to tumorigenesis, is unknown^48^. Therefore, the perinuclear region as a periphery of the core nucleus may serve as a restriction site for potentially oncogenic proteins such as Ras or as was shown recently for Src^8^ or for the regulation of tumor suppressor proteins such as p53. In these experiments Src was localized exclusively in the perinuclear region. Therefore, the perinucleus may play a genomic-protective role that impedes malignant cellular transformation; more detailed analysis and work is required in this new arena.

A specific extraction of nuclear pore complex proteins, such as Nup358, Nup98, and LINC proteins Nesprin-3, and partial extraction of Plectin-1 extend from the cytoplasmic side and Nup153 and partial extraction of Sun-2 extends from nuclear side to perinuclear fraction. This points to the existence of topological borders of the perinuclear region, which we formally call the *perinucleus*. This is not inconsistent with the previous work of Ketema and Sonnenberg^25^, who demonstrated that a perinuclear infrastructure includes nesprins and plectins that form, in part, an indirect protein link of the nucleus to the plasma membrane.

**Figure 6:**
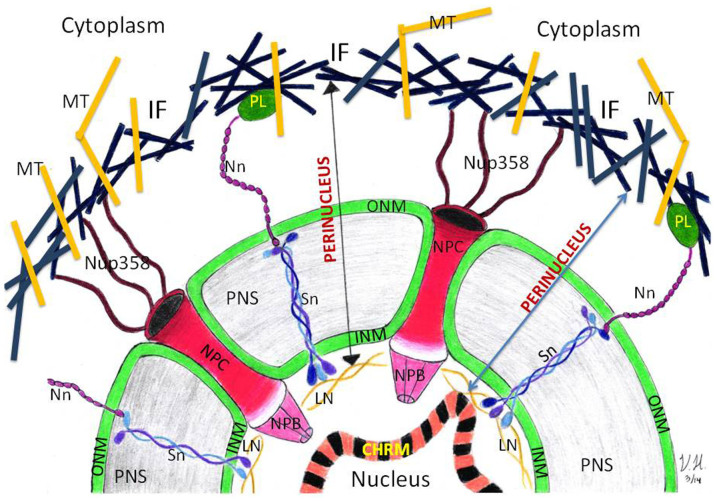
Model view of the perinucleus of cell. The nuclear boundary of the perinucleus is limited with the nuclear lamina where the N termini of SUN1 and SUN2 form a network with the nuclear lamina. The C termini of SUN proteins interact with the KASH domains of nesprins, that are located at the outer nuclear membrane, where it conntects to plectins (LINC complex). Plectin interacts with intermediate filaments, microtubules and the actin cytoskeleton. This filamentous region on the cytoplasmic side represents the outer boundary of the perinucleus. Note that the Sun 2 proteins form the nuclear boundaries and Plectin 1, from the cytosolic borders, were partially distributed in PNF, representing delimiting boundaries of the perinucleus. The core nucleus embedded into nucleoplasm, and supported by rigid lamin structure, does not collapse after extracting the perinucleus by CDS technique. KEY: CHRM – *chromatin*, IF – *intermediate filaments*, INM – *inner nuclear membrane*, LN – *lamin*, MT – *microtubules*, NPC – *nuclear pore complex*, Nn – *Nesprin proteins*, ONM – *outer nuclear membrane*, PL – *plectin proteins*, PNS – *perinuclear space*, Sn – *Sun proteins*.

The nuclear lamina was defined as a fibrous structure lining the inner nuclear membrane (INM), which is resistant to detergent and salt extraction^31^,^49^. In our experiment, Sun-2 was equally distributed in PNF and cNF, and Lamin B was not extracted to the PNF, but remained in the nucleus. Collectively, this indicates that the nuclear lamina is able to hold the integrity of the core nucleus or nucleoplasm together during fractionation. This experiment suggests a conceptual model in which the topological borders of the perinucleus can be stretched from the nuclear lamina from the nuclear side and the Plectin-Nesprin LINC complex, which is held in place by Sun proteins, from the cytoplasmic side (Figure 6). We delimited boundaries of the perinucleus from nuclear side as lamina because lamin B was not extracted to the PNF, but Sun 2 was equally distributed to the core nuclear and perinuclear fractions. Considering that Sun proteins form doublet and triplet structures themselves, in which some of proteins interact with nuclear lamin proteins, their partial distribution can be explained by the disassociation of Sun proteins. From the cytoplasmic side we delimited boundaries of perinucleus with filamentous region of cytoplasm since Plectin 1 was also partially extracted to the perinucleus. Taken together, the nuclear boundary of the perinucleus is limited within the nuclear lamina where the N termini of SUN-1 and SUN-2 form an interactive network with the nuclear lamina^50^.

The C termini of SUN proteins, residing in the nuclear envelope lumen^51^, interact with the KASH domains of nesprins located at the outer nuclear membrane where it connects through plectins (LINC complex) to intermediate filaments, microtubules and the actin cytoskeleton. This filamentous region on the cytoplasmic side together with nuclear pore complex and other protein complexes (Figure 2,3,4,5, panels B,C, and D) including the content of the perinuclear space represent the perinucleus, which protects and regulates the function of the genome embedded within the nucleoplasm. The nucleus with chromatin embedded into nucleoplasm and supported by rigid lamin structure and does not collapse after extracting the perinucleus by the CDS technique (Figure 1C), which represents the core nucleus (resistant to detergent and salt extractions)^49^.

In conclusion, the perinuclear complex, located between the cytoplasm and the nucleoplasm, could be a site for signal transmission where the aberrant cytoplasmic-signals are pre-empted^4^ and the normal signals are converted to nuclear action. It is possible that in malignant cell transformation, the signal transmission by the perinucleus becomes abnormal. Our method of perinucleus isolation would help to understand the mechanisms of the maintaining of genome integrity that ultimately should help the understanding of the mechanisms of malignant transformation.

## Methods

### Cell lines and reagents

MDA-MB-435 and HeLa cells were obtained from the American Type Culture Collection. RanBP2 haploinsufficient MEF cells were a gift from Dr. J. M. van Deursen^52^. Primary Mouse Embryonic Fibroblasts (pMEF) were isolated and cultured from the embryos of 13.5 days pregnant mice by standard procedure. Cells were grown to 95% confluent, in a media containing 10% FBS at 37°C, 5% CO_2_ for 48 hrs. Reagents were obtained from the following sources: DMEM/F12 from Life Technologies, the Fetal Bovine Serum (FBS) from Hyclone. Detergent compatible protein assay kit (DC Protein assay kit I #500-0111) was purchased from Bio Rad (Hercules, CA, USA). Antibodies: ERp57 (cat#05-728) was purchased from EMD Millipore (Billerica, MA, USA), BCL10 (sc-5273), Lamin B (sc-6216), RanBP2 (sc-15442), Nup153 (sc-292438; sc-20590), p53 (sc-6243), PML (sc-377303), Coilin* (sc-56298, sc-5594), B23 (sc-271737), α-Tubulin (sc-8035), COPE (sc-133194) were purchased from Santa Cruz Biotech. Inc. (Santa Cruz, CA, USA); CBP (cat# 4772), GM130 (cat# 2296), ERK42/44 (cat# 4695), PLD1 (cat# 3832), Plectin-1 (cat# 12254), Src (cat# 2123), Stat-3 (cat# 9139), PI3K p110α (cat# 4255), PTEN (cat# 9552), CREB (cat# 9197), Nup98 (cat# 2598), eIFα (cat# 9722), pan-Ras (cat# 3965), JunB* (cat# 3746), FOXC1* (cat# 7415) were purchased from Cell Signaling Technology (Danvers, MA, USA); Calnexin* (cat# 610523) was purchased from BD Transduction Labs. (San Jose, California USA); Mitofilin (ab137057), Sun 2 (ab124916), Calreticulin* (ab549220), Histone H3 (ab1791) were purchased from Abcam (Cambridge, MA, USA). Nesprin-3 (GTX87974) was purchased from GeneTex (Irvine, CA, USA). PKCα (p16520) was purchased from Transduction Labs (Lexington, KY, USA). HRP-labeled anti-rabbit/anti-mouse/anti-goat secondary antibodies were purchased from Santa Cruz Biotechnology (Santa Cruz, CA, USA). *Antibody does not react with mouse proteins.

Buffers:

1. Buffer A: 40 mM Hepes pH7.4, 120 ml KCl, 2 mM EGTA, 0.4% Glycerol, 10 mM β-glycerophosphate and 0.4% NP-40 with protease inhibitors.
2. Buffer B: 10 mM Tris-HCl pH 7.4, 1.5 mM KCl, 0.5% Triton X-100; 0.5% Deoxycholate, 2.5 mM MgCl_2_, with fresh 0.2 M LiCl and protease inhibitors.

### Cellular dissection method

Cells were lysed in isotonic buffer A with the final, critical concentration of the detergent in the lysate equal to 0.2% (approximately 0.3 ml of buffer A for cells growing in 15 cm dish) while rotating for 30 min at 4°C. Nuclei were pelleted by centrifugation at 1000 × g for 5 min; the supernatant was centrifuged further at 10,000 × g for 10 min to obtain the cytosolic fraction. The pellet of nuclei was sequentially and gently washed with 0.1% NP-40 and no-detergent-containing Buffer A and centrifuged at 1000 × g for 5 min; the supernatants were discarded. The nuclear pellet was re-suspended in Buffer B (ratio 1:2 v/v) and rotated for 1 hour at 4°C; the extract was separated by centrifugation at 2000 × g for 5 min. The extract was centrifuged further at 10,000 × g for 10 min to obtain the perinuclear fraction (PNF). Pelleted at 2000 × g, the core nuclei were resuspended in a buffer containing 0.34 M sucrose and separated by centrifugation at 2000 × g for 10 min. Nuclei were dissolved in 8 M urea, sonicated to obtain the core Nuclear Fraction (cNF), and clarified by centrifugation at 10,000 × g for 10 minutes.

### Nuclei isolation by using hypotonic buffer

To prepare nuclei, cells were swelled in a RBS buffer (10 mM Tris-HCl, 10 mM NaCl, 5 mM Mg acetate pH 7.4) per gram of cell pellet and allowed to swell on ice for 30 minutes. NP-40 detergent was added in final concentration 0.3% to the cell suspension and the mixture was homogenized with the Dounce tissue grinder using 50 strokes. The homogenate was centrifuged at 1200 × g for 10 min and the sedimented crude nuclei were resuspended in 20 volumes of 0.88 M sucrose with 5 mM Mg acetate and centrifuged at 2000 × g for 20 minutes. Nuclei were resuspended in 0.34 M sucrose with 5 mM Mg acetate and the recovered nuclei^53^ were dissolved in 8 M urea, sonicated, and clarified by centrifugation at 10,000 × g for 10 minute.

### Western blot analysis

The protein lysates were separated by 4%–15% SDS-PAGE and electrophoretically transferred to a PVDF (polyvinylidene difluoride) membrane (Millipore). The membrane was incubated with primary antibodies and then was incubated with HRP-labelled corresponding source of the secondary antibody. Subsequently, the proteins were detected by Immobilon chemiluminescent western blot reagents (Millipore).

## Author Contributions

T.E.S. conceived of, designed, performed the experiments and analyzed the data. A.R.O. and T.E.S. wrote the manuscript.

## Supplementary Material

Supplementary Information Supporting Documentation

## Figures and Tables

**Table 1:** Per cent of the total extracted proteins by Cell Dissection technique

Fraction	Buffers	MDA-MB435 Protein, %	HeLa Protein, %	MEF Protein, %	pMEF Protein, %
Cytosol	Buffer A	69.59 ± 0.19	69.89 ± 0.31	65.81 ± 0.63	74.28 ± 0.98
PNF	Buffer B	17.82 ± 0.09	15.22 ± 0.06	15.51 ± 0.09	12.0 ± 0.85
cNF	8 M Urea	12.60 ± 0.11	14.89 ± 0.26	18.67 ± 0.54	13.72 ± 0.35

PNF: perinuclear fraction, cNF: core nuclear fraction.

**Table 2:** Subcellular localization of the investigated proteins

		*Cyt: cytosol; PN: perinucleus; Nuc: nucleus; Mt: mitochondrion*			
#	Proteins	MDA-MB-435	HeLa	MEF	pMEF
1	PI3K	Cyt	Cyt	Cyt	Cyt
2	ER57	Cyt	Cyt	Cyt	Cyt
3	Mitofilin	Cyt (Mt)	Cyt (Mt)	Cyt (Mt)	Cyt (Mt)
4	PTEN	Cyt > PN	Cyt	Cyt > PN	Cyt > PN
5	Tubulin	Cyt > PN	Cyt	Cyt	Cyt
6	Calreticulin	Cyt, PN	Cyt	-	-
7	CBP	Cyt, PN	Cyt, PN	PN	PN
8	Calnexin	Cyt, PN	Cyt, PN	-	-
9	eIFα	Cyt, PN	Cyt, PN	Cyt, PN	Cyt, PN
10	COPE	Cyt, PN	Cyt	Cyt	Cyt
11	P53	Cyt, PN	PN	Nuc, PN	PN
12	RBP2	PN	PN	PN	PN
13	Nup153	PN	PN	PN	Cyt, PN
14	GM130	PN	PN	PN	Cyt, PN
15	Ras	PN	PN	Nuc, PN	PN
16	PML	PN, Nuc	PN	-	-
17	Coilin	Nuc	Nuc	-	-
18	B23	Nuc	Nuc	Nuc, PN	Nuc
19	Histone H3	Nuc	Nuc	Nuc	Nuc
20	CREB	Nuc, PN	Nuc, PN	Nuc, PN	Nuc, PN
21	JunB	Nuc, PN	PN	-	-
22	FOXC1	Nuc, PN	PN	-	-
23	c-Src	PN	PN	PN	PN
24	STAT3	Cyt, PN	All fraction	Cyt, PN	Cyt, PN
25	PKC alpha	Cyt	Cyt	Cyt	Cyt
26	Plectin1	Cyt, PN	Cyt, PN	Cyt, PN	Cyt
27	PLD1	Cyt, PN	Cyt, PN	All fraction	All fraction
28	BCL10	Cyt	Cyt	Cyt	Cyt
29	ERK 1,2	Cyt, PN	Cyt	Cyt, PN	Cyt, PN
30	Nup98	PN	PN	PN, Nuc	PN, Nuc
31	Sun 2	PN, Nuc	PN	PN, Nuc	PN, Nuc
32	Nesprin-3	PN, Nuc	PN	PN	PN
33	Lamin B	Nuc	Nuc	Nuc	Nuc
