# Author Correction: Dissecting the cell to nucleus, perinucleus and cytosol

**DOI:** 10.1038/s41598-021-88968-0

**Published:** 2021-04-22

**Authors:** Tattym E. Shaiken, Antone R. Opekun

**Affiliations:** 1 Department of Molecular and Cellular Oncology, University of Texas M. D. Anderson Cancer Center, Houston, TX 77030 USA; 2 Departments of Medicine & Pediatrics G.I. & S.A.H.S. Baylor College of Medicine-McNair Faculty Center A10.019 One BaylorPlaza (GI Medicine MS901), Houston, TX 77030 USA

Correction to: *Scientific Reports* 10.1038/srep04923, published online 12 May 2014.

This Article contains a typographical error in the protocol for Buffer A in KCl concentration where,

“120 ml KCl”

should read:

“120 mM KCl”

Additionally, the corresponding email of Tattym Shaiken is incorrect. Correspondence and request for materials should be addressed to tattym3431@att.net.

