# Supplementary material for: Dissecting the cell to nucleus, perinucleus and cytosol
Source: Sci Rep. 2014 May 12;4:4923. doi: 10.1038/srep04923 (PMC4017230; doi:10.1038/srep04923)
Supplement: Supplementary Information — Supporting Documentation [file srep04923-s1.pdf]

Supplementary Information for

SREP-14-00818 revised

Dissecting the cell to nucleus, perinucleus and cytosol

Tattym E. Shaiken[1,*], Antone R. Opekun[2]

*To whom correspondence should be addressed. E-mail: tshaikenof@comcast.net

Experiment designing:

1. **Cells:**
   - MDA-MB-435
   - HeLa
   - SV40 large T antigen immortalized MEF cells
   - Primary MEF cells isolated from 13.5 pregnant mouse

   (Cell growth condition is described in Method section)

2. **Sample preparation and labeling:**
   - Control cytosol lysate was prepared using 0.3%Chaps lysis buffer
     (Marked as Cyt. Control in final figures and C in films)
   - Control nuclear lysate was prepared using 8M Urea
     (Marked as Nuc. Control in final figures and N in films)
   - Experimental samples were prepared with the use of new Cell dissection technique
     (Marked as Cyt for cytosol, PeriNuc for perinucleus and cNuc for core nuclear fraction in final figures and C, P, and N (orcN) in films correspondingly)

3. **Sequence of sample loading in the 4-15% gradient PAGE gel**

1	2	3	4	5	6
marker	Cyt.Control	Cyt	PeriNuc	cNuc	Nuc.Control
In films:	C	C	P	N	N
Sample prep:	0.3%Chaps lysate	Cell dissection technique			Hypontonic Nuclei isolation

4. **Western blot Analysis**
   Unless otherwise stated the membranes were cropped into two halves and marked top halve as T and coded as follows: AT&Ae is the same membrane cropped into two halves and so on.

Localization – Cellular localization; MW –molecular weight of protein, Source – Antibody source

#	Loca-lization	MW	1st Antibody	Labeling	Source
1	PN	358	RBP2	AT	Goa
2	PM	47	PTEN	Ae	Rab
3	Cyt	38	eIFα	Be	Rab
4	Glg	130	GM130	BT	Rab
5	PM	21	Ras	CE	Rab
6	PM	110	PI3K	**CT**	Rab
7	VS	36	COPE	De	Mo
8	N	80	Coilin	DT	Mo
9	ER	90	Calnex	ET	Mo
10	N	43	CREB	Fe	Rab
11	N	300	CBP	FT	Rab
12	PN	153	Nup153	GT	Goa
13	N	42	B23	Ke	Mo
14	N	32	Hist3	Ee	Rab
15		106	PML	GT	Mou

#	Loca-lization		1st Antibody	Labeling	Source
1	PN	358	RBP2	LT	Goa
2	N	300	CBP	MT	Rab
3	N	153	Nup153	NT	Rab
4	Glg	130	GM130	OT	Rab
5	Vlt	110	LRP	PT	mo
6	PM	110	PI3K	RT	Rab
7	N	106	PML	ST	mou
8	PN	98	Nup98	UT	Rab
9	ER	90	Calnex	WT	mou
10	Mit	84	Mitofilin	XT	Rab
**11**	N	80	Coilin	L	mo
12	ER	57	ER57	M	mou
13	ER	55	Calreticulin	N	Mou
14	N	53	P53	O	rab
15	Cyt	50	Tubulin	P	mou
16	PM	47	PTEN	R	Rab
17	N	43	CREB	S	Rab
18	N	42	B23	U	mo
19	Cyt	38	eIFα	W	Rab
20	VS	36	COPE	X	mo

Examples of the PVDF membrane cropping and blotting:

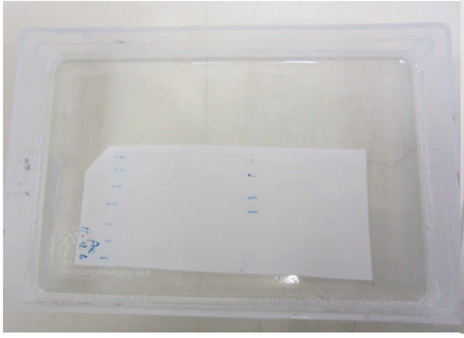
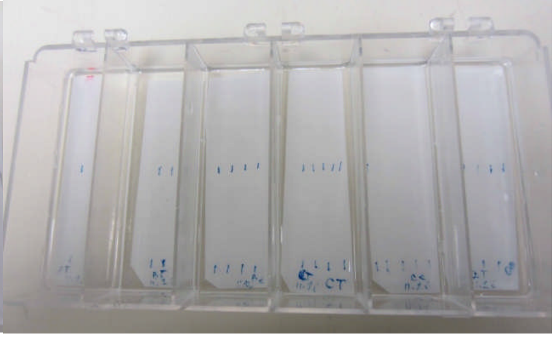

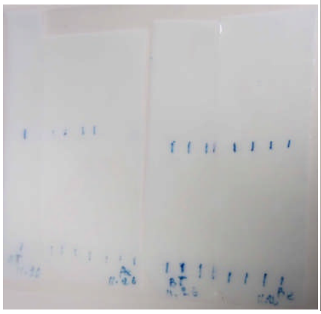
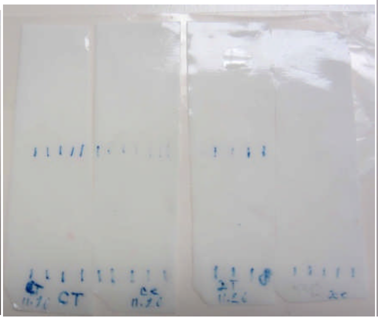
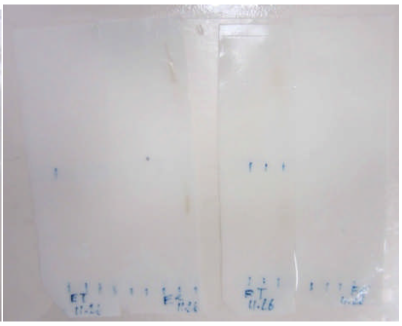

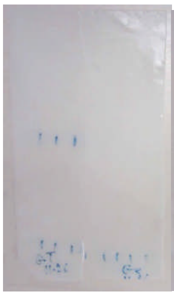
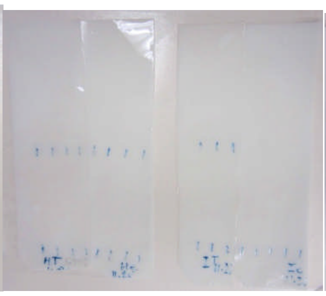
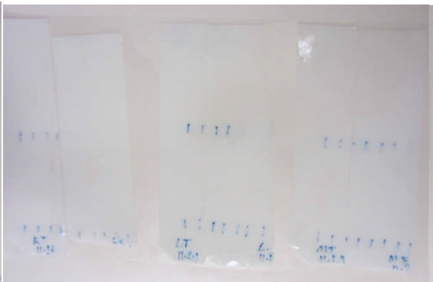
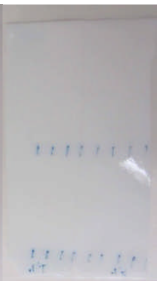

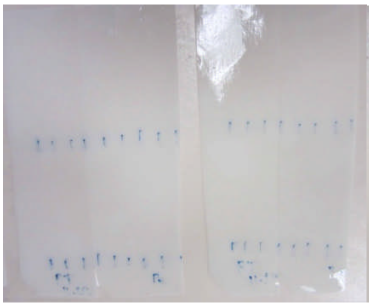
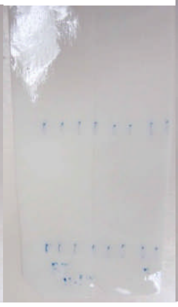
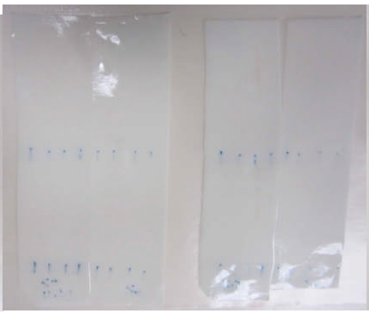
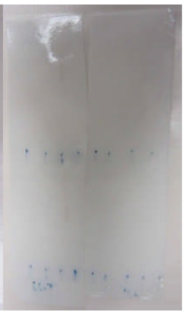

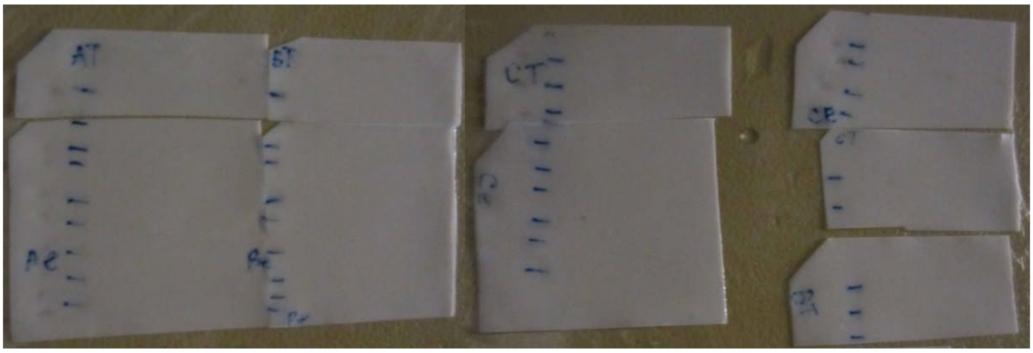

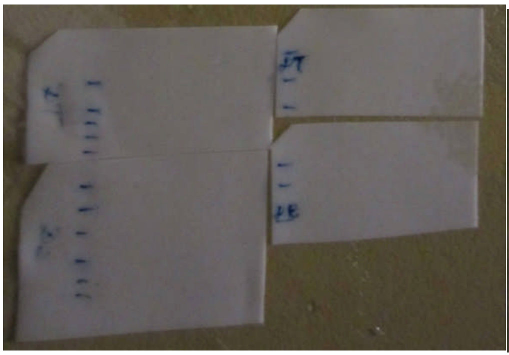

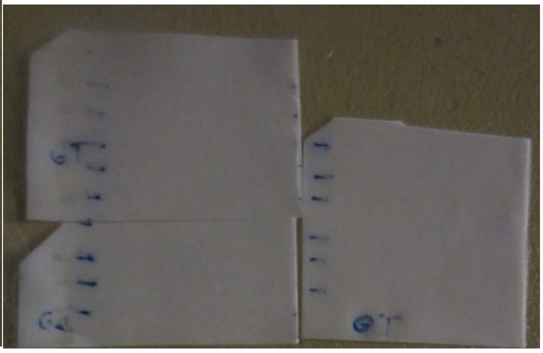

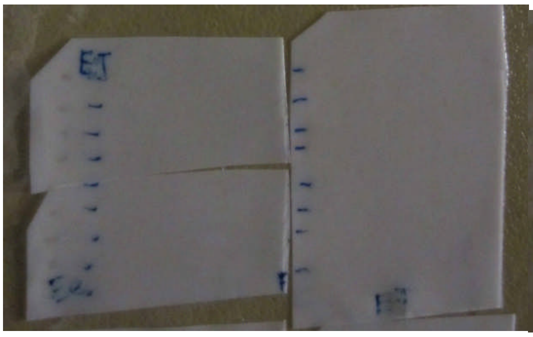

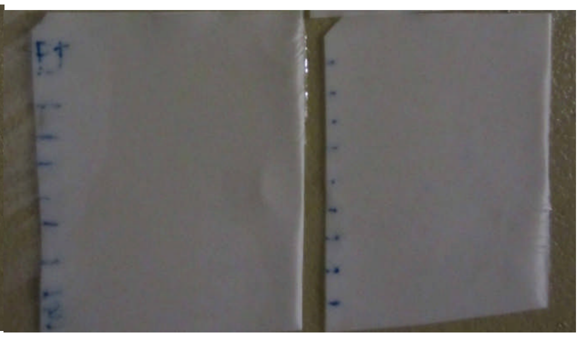

Added on March 22, 2014

1. **Western blot Analysis**
   The membranes were cropped into three segments: the top segment was cut above 150 kDa protein marker and used for detection of Plectin-1 which has the mol weight about 500 kDa; the medium and the bottom segments were obtained by cutting on the line of 75 kDa protein marker, that were used to detect low and medium mol weight proteins correspondingly.

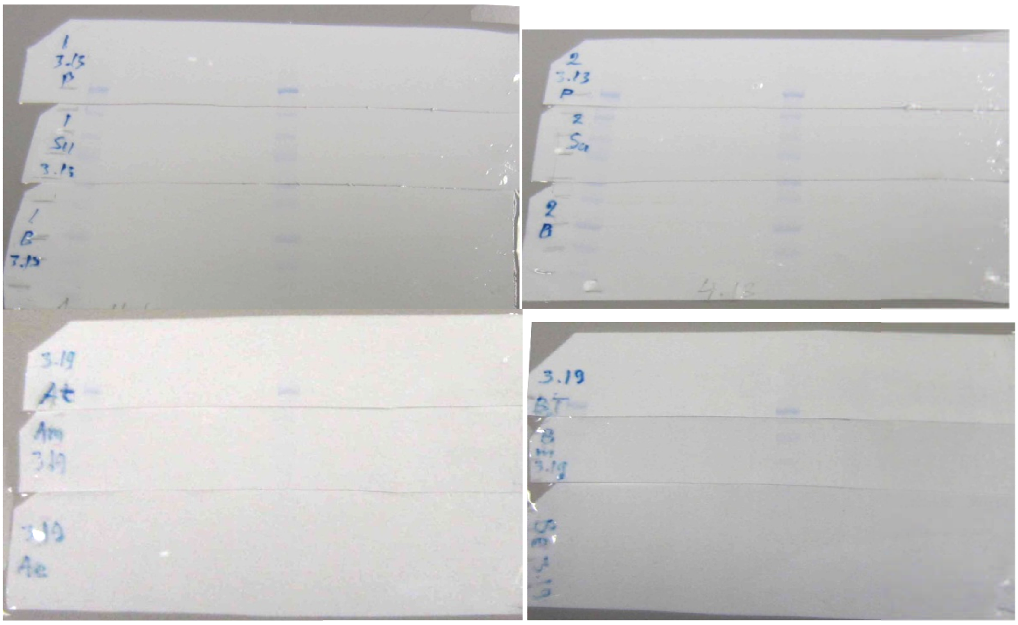

5. Protein visualization

After incubation with the corresponding source of secondary Antibody proteins were visualized with ECL detection technique.

Protein bands are shown in an alphabetic order for the convenience

Figure 2.
A lower cropped part of membrane for B23 blotting; MDA-MB-435 cells

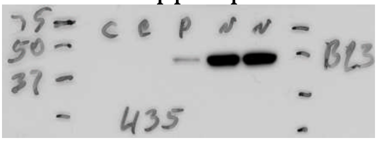

Figure 3.
A lower cropped part of membrane for B23 blotting; HeLa cells

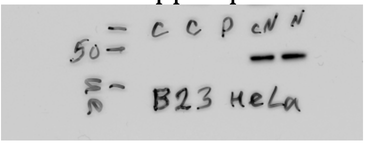

Figure 4.
A whole membrane for B23 blotting; MEF cells

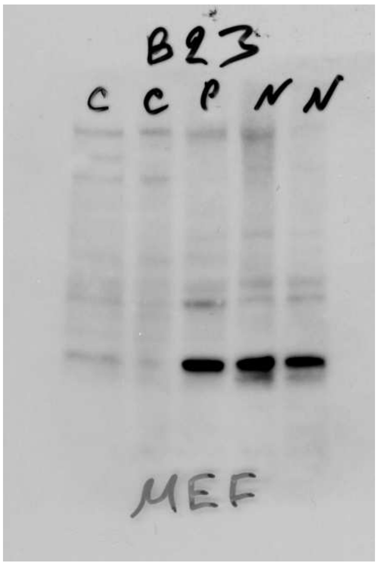

Figure 5.
A whole membrane for B23 blotting; primary MEF cells

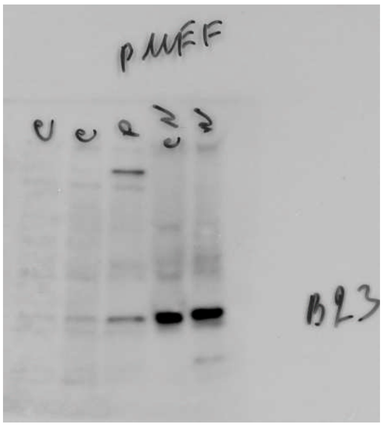

Note for B23 nucleophosmin detection

B23 is a nucleolar protein and its perinuclear detection only in MEF cells correlated with the p53 translocation to the perinucleus, which supports its stabilizing role for p53.

Figure 2.
An upper cropped part of membrane for Calnexin blotting; MDA-MB-435 cells

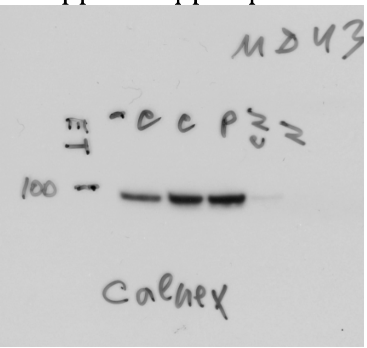

Figure 3.
An upper cropped part of membrane for Calnexin blotting; HeLa cells

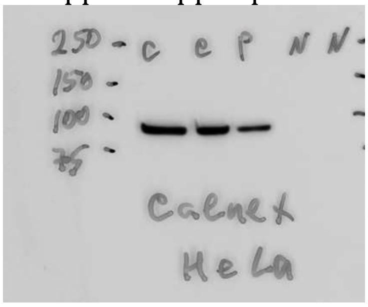

Note for Calnexin detection

Calnexin, an ER chaperone protein**,** appeared as a 90kDa band on western blotting, localized in cytosole and perinuclear region as well. Anti-Calnexin antibody does not crossreact with mouse protein.

Figure 2.
A lower cropped part of membrane for Calreticulin blotting; MDA-MB-435 cells

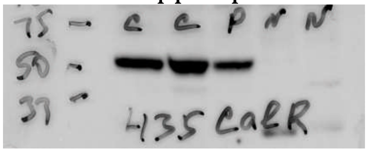

Figure 3.
A lower cropped part of membrane for Calreticulin blotting; HeLa cells

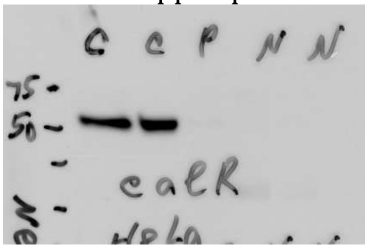

Note for Calreticulin detection

Calreticulin is Ca^{++} ions binding ER protein appeared only in the cytosol fraction detected with both Chaps lysis and CDS methods. Anti- Calreticulin antibody does not crossreact with mouse protein.

Figure 2.
An upper cropped part of membrane for CBP blotting; MDA-MB-435 cells

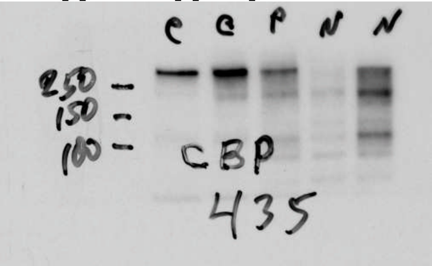

Figure 3.
An upper cropped part of membrane for CBP blotting; HeLa cells

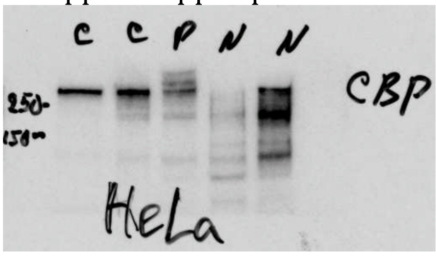

Figure 4.
An upper cropped part of membrane for CBP blotting; MEF cells

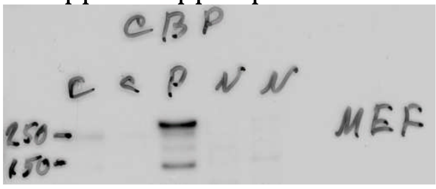

Figure 5.
An upper cropped part of membrane for CBP blotting; primary MEF cells

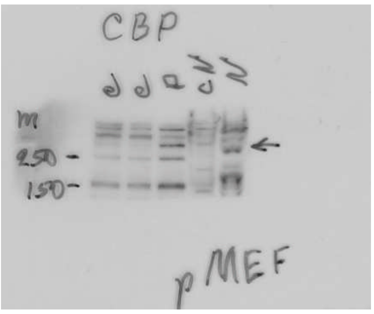

Note for CBP detection

CBP, CREB binding protein detects in cytosol and perinuclear fractions of cancer cell lines MDA-MB-435 and HeLa cells, but it localizes in perinucleus of MEF cells

Figure 2.
An upper cropped part of membrane for coilin blotting; MDA-MB-435 cells

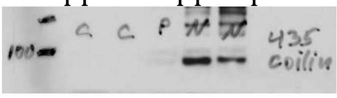

Figure 3.
An upper cropped part of membrane for coilin blotting; HeLa cells

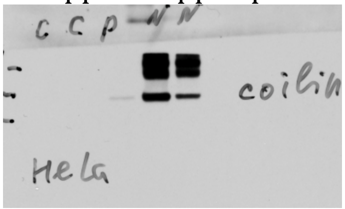

Note for Coilin detection
Coilin protein is one of the main molecular components of Cajal bodies. Cajal bodies are nuclear suborganelles. Coilin detects only in nuclear fraction with both nuclear isolation methods. Anti- Coilin antibody does not crossreact with mouse protein.

Figure 2.
A lower cropped part of membrane for COPE blotting; MDA-MB-435 cells

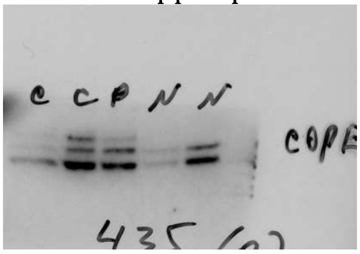

Figure 3.
A lower cropped part of membrane for COPE blotting; HeLa cells

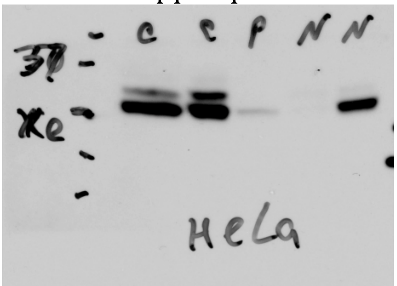

Figure 4.
A lower cropped part of membrane for COPE blotting; MEF cells

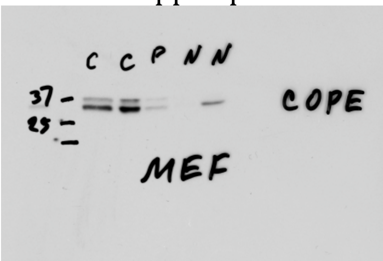

Figure 5.
A lower cropped part of membrane for COPE blotting; pMEF cells

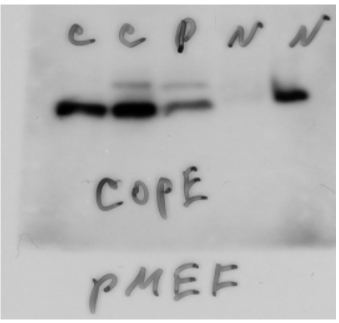

Note for COPE detection

COPE is epsilon subunit of coatomer protein complex. Coatomer is a cytosolic protein complex that binds to dilysine motifs and reversibly associates with Golgi non-clathrin-coated vesicles. COPE appears in cytosol and in perinucleus of MDA-MB-435 cells as several bands.

Figure 2.
A lower cropped part of membrane for CREB blotting; MDA-MB-435 cells

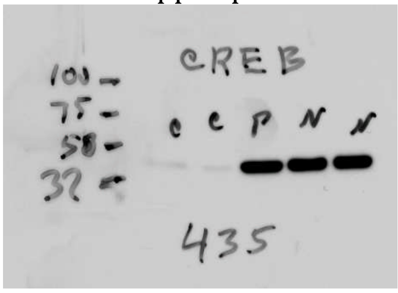

Figure 3.
A lower cropped part of membrane for CREB blotting; HeLa cells

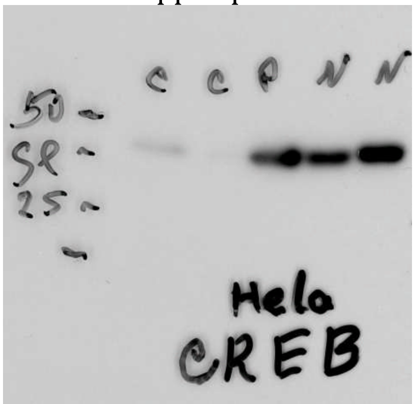

Figure 4.
A lower cropped part of membrane for CREB blotting; MEF cells

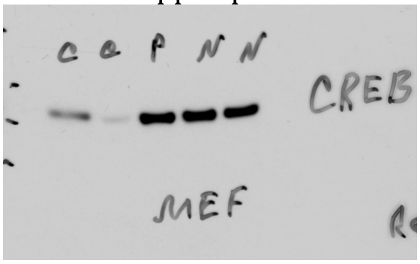

Figure 5.
A lower cropped part of membrane for CREB blotting; pMEF cells

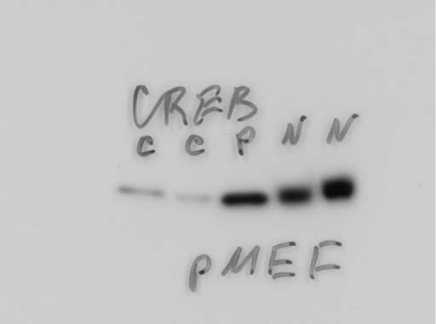

Note for CREB detection

CREB is a is a transcription factor detected in nucleus and perinucleus

Figure 2.
A lower cropped part of membrane for eIFα blotting; MDA-MB-435 cells

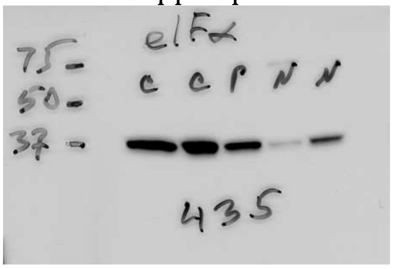

Figure 3.
A lower cropped part of membrane for eIFα blotting; HeLa cells

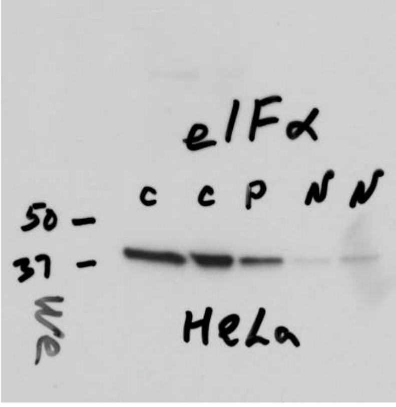

Figure 4.
A lower cropped part of membrane for eIFα blotting; MEF cells

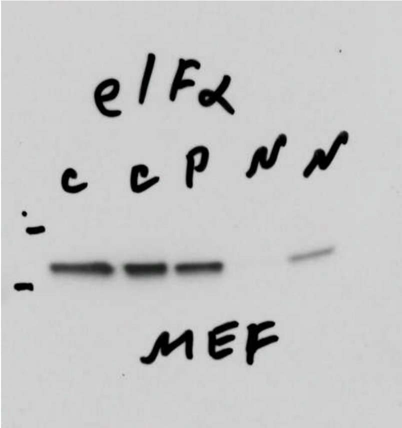

Figure 5.
A lower cropped part of membrane for eIFα blotting; pMEF cells

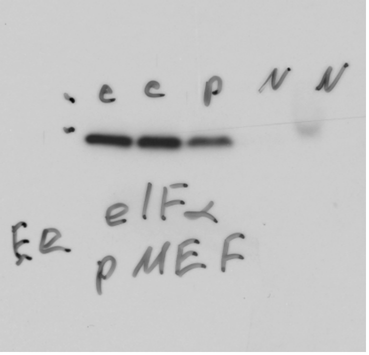

Note for eIFα detection

eIF2 is a heterotrimer consisting of an alpha, a beta, and a gamma subunit. Once the initiation is completed, eIF2 is released from the ribosome. Alpha subunit is detected in cytosol and perinucleus fractions, which suggests the protein synthesis initiation may start in the perinucleus.

Figure 2.
A lower cropped part of membrane for ERp57 blotting; MDA-MB-435 cells

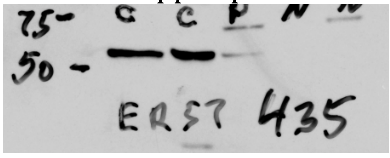

Figure 3.
A lower cropped part of membrane for ERp57 blotting; HeLa cells

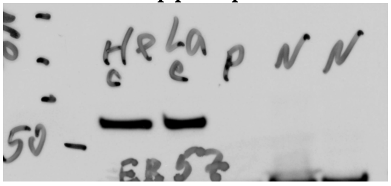

Figure 4.
A lower cropped part of membrane for ERp57 blotting; MEF cells

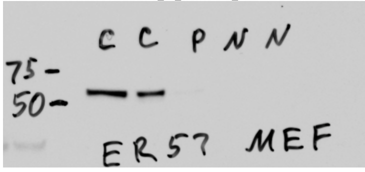

Figure 5.
A lower cropped part of membrane for ERp57 blotting; pMEF cells

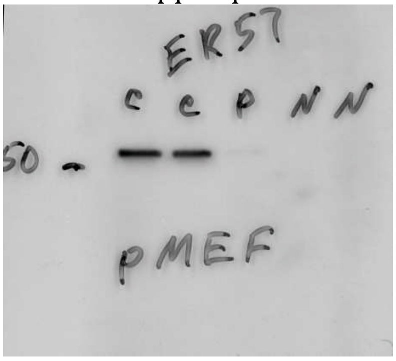

Note for ERp57 detection

ERp57 is a soluble protein of the endoplasmic reticulum and serves as ER marker, detected in cytosol fraction

Figure 2.
A lower cropped part of membrane for FOXC1 blotting; MDA-MB-435 cells

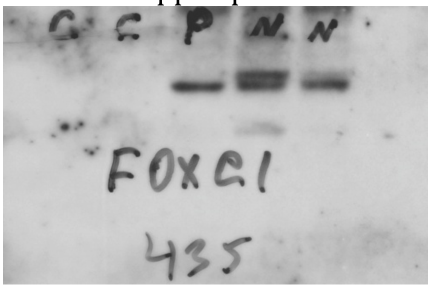

Figure 3.
A lower cropped part of membrane for FOXC1 blotting; HeLa cells

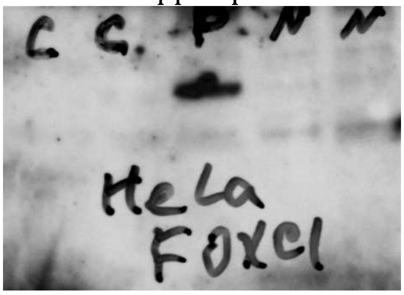

Note for FOXC1 detection

Forkhead box C1 protein is a transcription factor, has been shown to play a role in the regulation of embryonic and ocular development. In HeLa cells it localized in perinucleus but in MDA-MB-435 cells it is also detected in the nucleus. Nuclear FOXC1 has a slow moving component. Anti-FOXC1 antibody does not crossreact with mouse protein.

Figure 2.
An upper cropped part of membrane for GM130 blotting; MDA-MB-435 cells

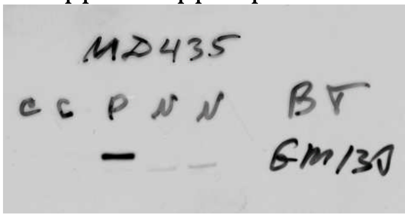

Figure 3.
An upper cropped part of membrane for GM130 blotting; HeLa cells

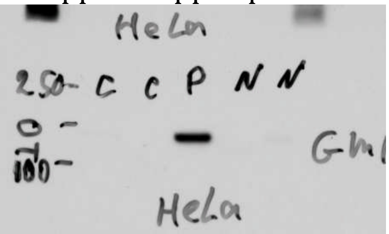

Figure 4.
An upper cropped part of membrane for GM130 blotting; MEF cells

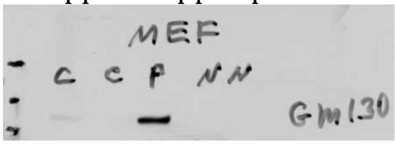

Figure 5.
An upper cropped part of membrane for GM130 blotting; pMEF cells

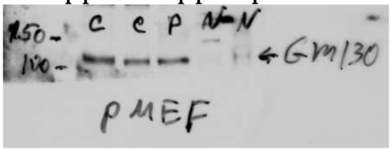

Note for GM130 detection

The Golgi matrix protein GM130 is a Golgi complex marker, detected in Perinucleus of cancer cell lines of MDA-MB-435 and HeLa cells and immortalized MEF but in normal cells it detected also in the cytosolic fraction.

Figure 2.
A lower cropped part of membrane for Histone H3 blotting; MDA-MB-435 cells

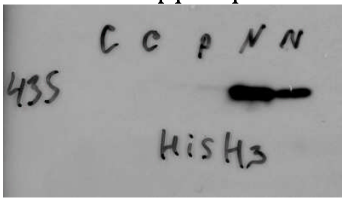

Figure 3.
A lower cropped part of membrane for Histone H3 blotting; HeLa cells

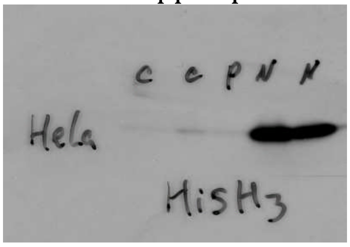

Figure 4.
A lower cropped part of membrane for Histone H3 blotting; MEF cells

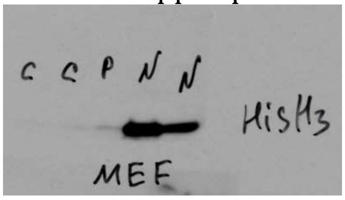

Figure 5.
A lower cropped part of membrane for Histone H3 blotting; pMEF cells

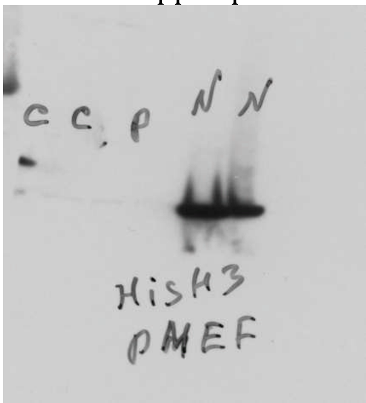

Note for Histone H3 detection

Histone H3 is involved in the structure of chromatin in eukaryotic cells. The light from the membrane was so strong that the main problem was to quench the signal coming from the nuclear fractions of the cell.

Figure 2.
A lower cropped part of membrane for JunB blotting; MDA-MB-435 cells

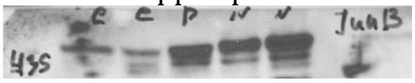

Figure 3.
A lower cropped part of membrane for JunB blotting; HeLa cells

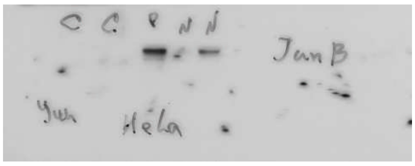

Note for JunB detection

Jun-B is a transcription factor involved in regulating gene activity following the primary growth factor response. It detected in perinuclear fraction of HeLa cells and in perinuclear and nuclear fractions of MDA-MB-435 cells. Anti-JunB antibody does not crossreact with mouse protein.

Figure 2.
An upper cropped part of membrane for Mitofilin blotting; MDA-MB-435 cells

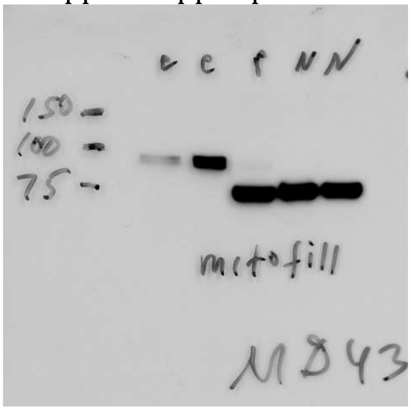

Figure 3.
An upper cropped part of membrane for Mitofilin blotting; HeLa cells

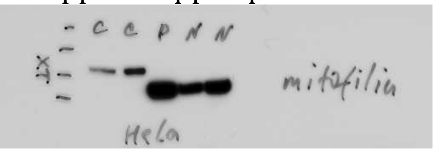

Note for mitofilin detection

The mitochondrial inner membrane protein mitofilin is detected in cytosol. Since mitochondrion does not exist in the nucleus the bands, which appeared in perinucleus and in the nucleus are non-specific. Anti-mitofilin antibody does not crossreact with mouse protein.

Figure 2.
An upper cropped part of membrane for Nup153 blotting; MDA-MB-435 cells

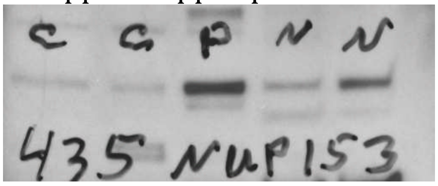

Figure 3.
An upper cropped part of membrane for Nup153 blotting; HeLa cells

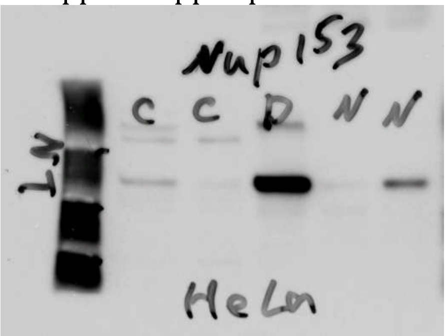

Figure 4.
An upper cropped part of membrane for Nup153 blotting; MEF cells

C C P N N

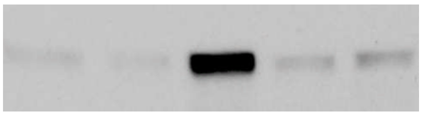

Figure 5.
An upper cropped part of membrane for Nup153 blotting; pMEF cells

C C P N N

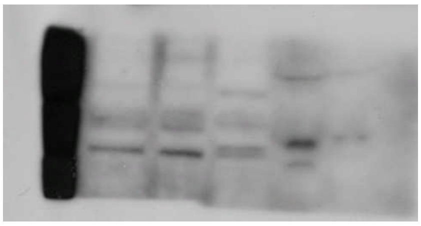

Note for Nup153 detection

The nuclear pore basket protein Nup153 localizes at the nuclear side of the nuclear pore complex. The protein was detected in perinucleus of the HeLa and MDA-MB-435 cancer cell lines and the immortalized MEF. It was detected in normal MEF cells at high exposure of the film.

Figure 2.
A lower cropped part of membrane for p53 blotting; MDA-MB-435 cells

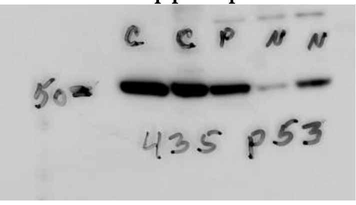

Figure 3.
A lower cropped part of membrane for p53 blotting; HeLa cells

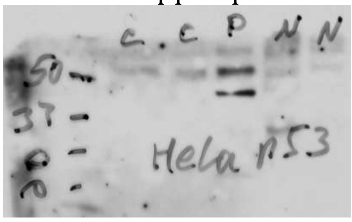

Figure 4.
A lower cropped part of membrane for p53 blotting; MEF cells

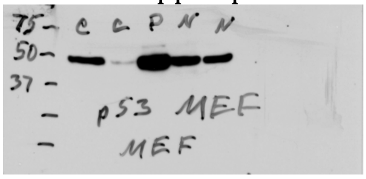

Figure 5.
A lower cropped part of membrane for p53 blotting; pMEF cells

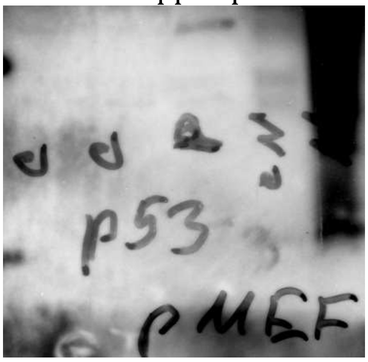

Note for p53 detection

Tumor suppressor p53 has been described as "the guardian of the genome" because of its role in conserving stability by preventing genome mutation. The protein was detected with high exposure of film in primary MEF and HeLa cells. Mutated p53 of MDA-MB-435 cells appeared in the cytosol and perinucleus. p53 of the SV40 large T antigen immortalized MEF cells localized in perinucleus and nucleus.

Figure 2.
An upper cropped part of membrane for PI3K 110α blotting; MDA-MB-435 cells

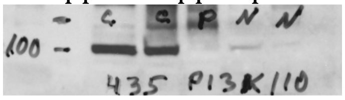

Figure 3.
An upper cropped part of membrane for PI3K 110α blotting; HeLa cells

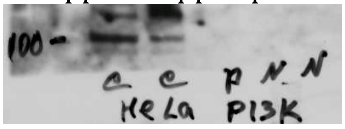

Figure 4.
An upper cropped part of membrane for PI3K 110α blotting; MEF cells

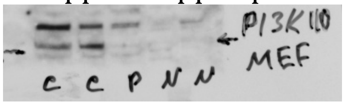

Figure 5.
An upper cropped part of membrane for PI3K 110α blotting; pMEF cells

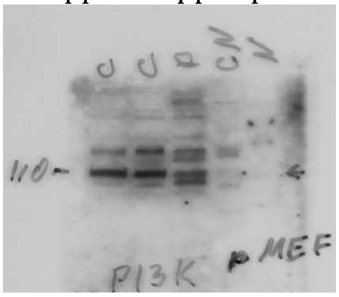

Note for PI3K 110 α detection

Class IA PI3K is composed of a heterodimer between a p110 catalytic subunit and a p85 regulatory subunit. The p110 catalytic subunit was detected in cytosol of HeLa and MDA-MB-435 cancer cell lines and the immortalized MEF. A double band of protein was detected in perinucleus of normal MEF cells.

Figure 2.
An upper cropped part of membrane for PML blotting; MDA-MB-435 cells

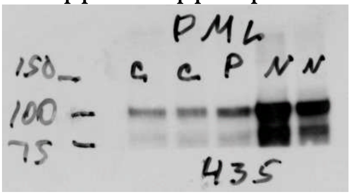

Figure 3.
An upper cropped part of membrane for PML blotting; HeLa cells

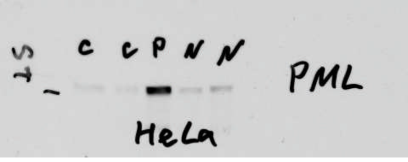

Figure 4.
A whole membrane for PML blotting; MEF cells

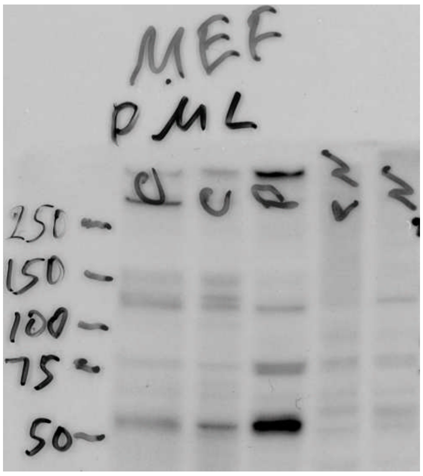

Figure 5.
A whole membrane for PML blotting; pMEF cells

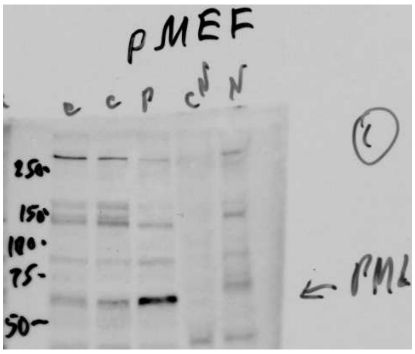

Note for PML detection

The Promyelocytic leukemia protein (PML) is found in distinct structures in the nucleus of a cell called PML nuclear bodies (PML-NBs). A variety of PML proteins ranging in molecular weight from 48-97 kDa were detected[1]. HeLa and MDA-MB-435 cancer cell lines it showed high molecular weight bands however in MEF cells the protein appeared at approximately 50 kDa molecular weight range. PML was detected in the perinuclear and nuclear fractions of the investigated cells.

Figure 2.
A lower cropped part of membrane for PTEN blotting; MDA-MB-435 cells

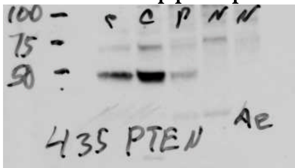

Figure 3.
A lower cropped part of membrane for PTEN blotting; HeLa cells

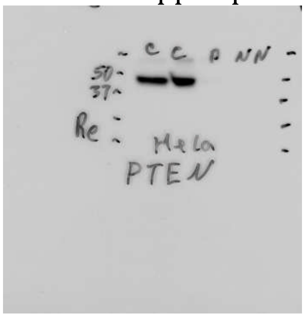

Figure 4.
A lower cropped part of membrane for PTEN blotting; MEF cells

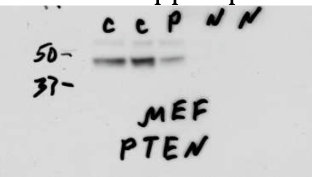

Figure 5.
A lower cropped part of membrane for PTEN blotting; pMEF cells

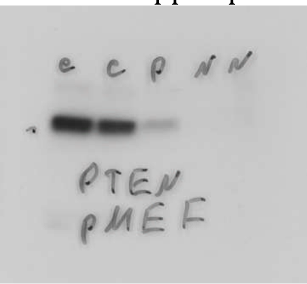

Note for PTEN detection

PTEN, a phosphatase, is involved in the regulation of the cell cycle, preventing cells from growing and dividing too rapidly. The protein was detected mainly in cytosol, but a minor band was also found in the perinucleus.

Figure 2.
A lower cropped part of membrane for RanBP2 blotting; MDA-MB-435 cells

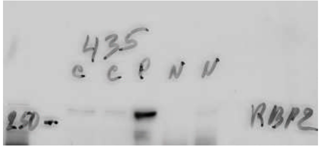

Figure 3.
An upper cropped part of membrane for RanBP2 blotting; HeLa cells

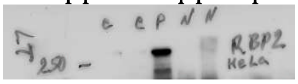

Figure 4.
An upper cropped part of membrane for RanBP2 blotting; MEF cells

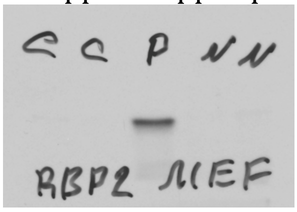

Figure 5.
An upper cropped part of membrane for X blotting; pMEF cells

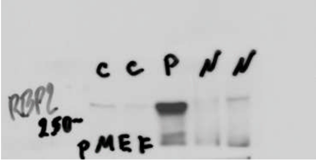

Note for RanBP2/Nup358 detection

RanBP2 forms the cytoplasmic filaments of the nuclear pore complex. The pleiotropic role of RanBP2 reflects its interaction with multiple partners, each presenting distinct cellular or molecular functions. The protein was detected in perinucleus.

Figure 2.
A lower cropped part of membrane for Ras blotting; MDA-MB-435 cells

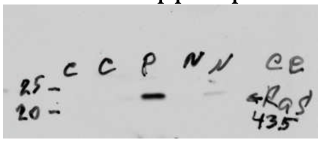

Figure 3. A lower cropped part of membrane for Ras blotting; HeLa cells

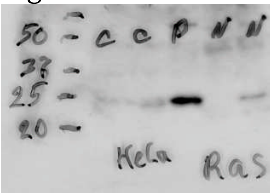

Figure 4.
A lower cropped part of membrane for Ras blotting; MEF cells

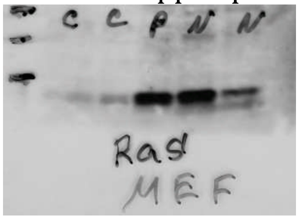

Figure 5.
A lower cropped part of membrane for Ras blotting; pMEF cells

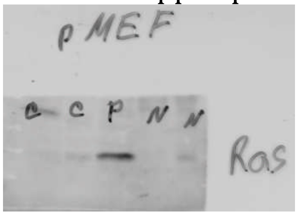

Note for Ras detection

All Ras protein family members belong to a class of protein called small GTPase, and are involved in transmitting signals within cells. Targeting of Ras isoforms to plasma membrane have been implicated in Ras signaling and oncogenic potential[2]. However, Kras alone might not be sufficient to transform a cell. Numerous studies reported that healthy people have cells with oncogenic K-Ras in different organs, at rates far exceeding the rates of cancer development[3]. In this experiment Ras protein was detected only in perinucleus, which suggests that the protein is probably not in an active form.

Figure 2.
A whole membrane for a-Tubulin blotting; MDA-MB-435 cells

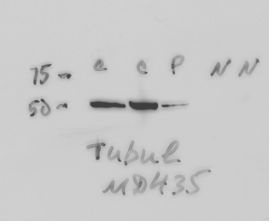

Figure 3.
A lower cropped part of membrane for a-Tubuline blotting; HeLa cells

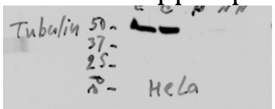

Figure 4.
A lower cropped part of membrane for a-Tubuline blotting; MEF cells

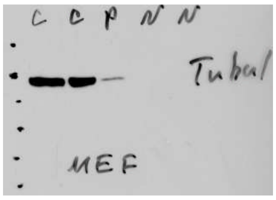

Figure 5.
A lower cropped part of membrane for a-Tubuline blotting; pMEF cells

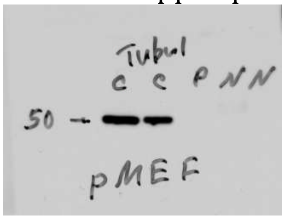

Note for α–Tubulin detection
α-Tubulin and β-tubulin are most common proteins that make up microtubules.
Tubulin was detected predominantly in cytosol fraction.

Figure 2.
A medium segment of membrane was used for Lamin B blotting; MDA-MB-435 cells

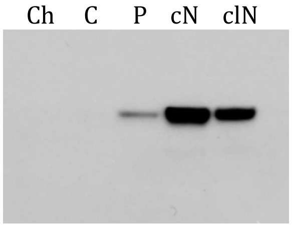

Figure 3.
A medium segment of membrane was used for Lamin B blotting; HeLa cells

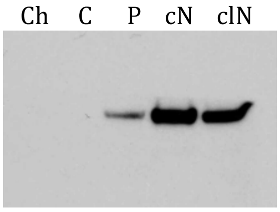

Figure 4.
A medium segment of membrane was used for Lamin B blotting; MEF cells

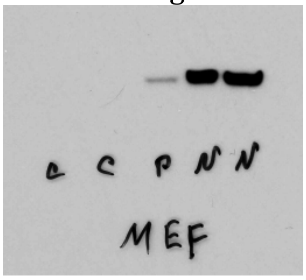

Figure 5.
A medium segment of membrane was used for Lamin B blotting; pMEF cells

Ch C P cN clN

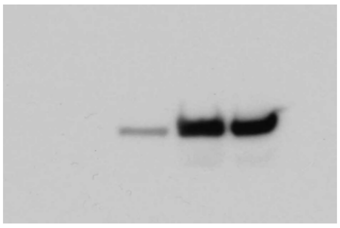

Note for Lamin B detection

The nuclear lamina lies on the inner surface of the inner nuclear membrane (INM), where it serves to maintain nuclear stability, organize chromatin and bind nuclear pore complexes (NPCs). B-type lamins are present in every cell and part of nuclear lamina. Lamin B was detected in nuclear fraction.

Figure 2.
A medium segment of membrane was used for Nup98 blotting; MDA-MB-435 cells

Ch C P cN clN

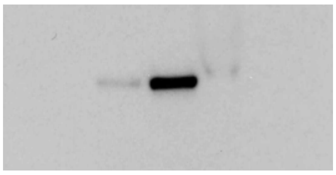

Figure 3.
A medium segment of membrane was used for Nup98 blotting; HeLa cells

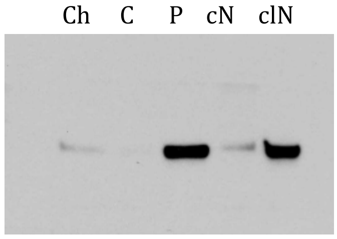

Figure 4.
A medium segment of membrane was used for Nup98 blotting; MEF cells

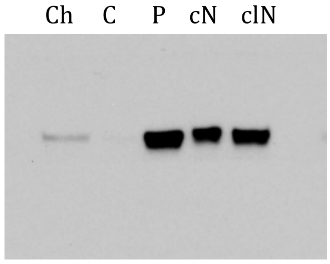

Figure 5.
A medium segment of membrane was used for Nup98 blotting; pMEF cells

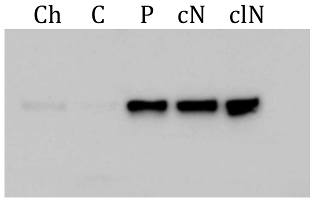

Note for Nup98 detection

Nup98 is a 98 kD nucleoporin, nuclear pore complex (NPC) protein, localized to the nucleoplasmic side of the NPC. CDS method is able to extract this protein to perinuclear fraction, however in MEF cells part of the Nup98 is bound to the nucleus after extraction.

Figure 2.
A medium segment of membrane was used for Src blotting; MDA-MB-435 cells.

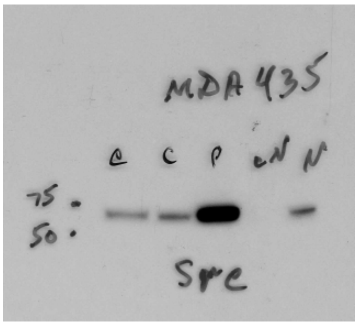

Figure 3.
A medium segment of membrane was used for Src blotting; HeLa cells.

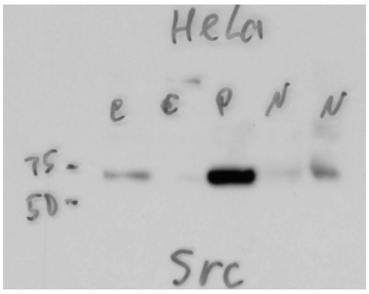

Figure 4.
A medium segment of membrane was used for Src blotting; MEF cells

Ch C P cN clN

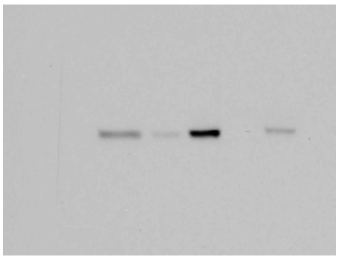

Figure 5.
A medium segment of membrane was used for Src blotting; pMEF cells

Ch C P cN clN

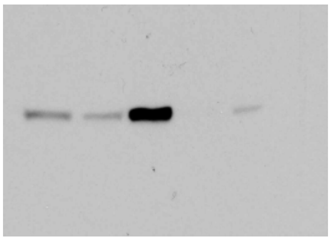

Note for Src detection

c-Src is a non-receptor protein tyrosine kinase. Reinecke J.B. et al (2014) demonstrated that inactive Src is localized in the perinuclear endocytic recycling compartment. Src was detected in the perinuclear fraction.

1. Jensen, K., Shiels, C. & Freemont, P.S. PML protein isoforms and the RBCC/TRIM motif. *Oncogene* **20**, 7223-7233 (2001).
2. Eisenberg, S. & Henis, Y.I. Interactions of Ras proteins with the plasma membrane and their roles in signaling. *Cellular signalling* **20**, 31-39 (2008).
3. di Magliano, M.P. & Logsdon, C.D. Roles for KRAS in pancreatic tumor development and progression. *Gastroenterology* **144**, 1220-1229 (2013).

For suggestions of Reviewer 1:

Figure 2.
A top segment of membrane was used for Plectin-1 blotting; MDA-MB-435 cells.

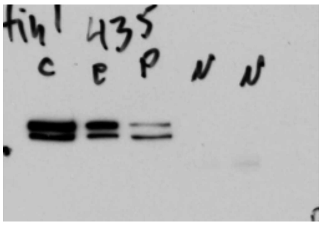

Figure 3.
A top segment of membrane was used for Plectin-1 blotting; HeLa cells.

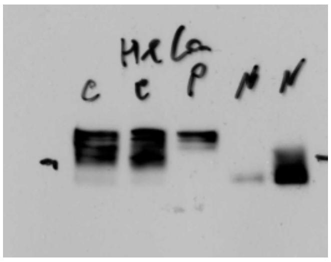

Figure 4.
A top segment of membrane was used for Plectin-1 blotting; MEF cells.

Ch C P cN clN

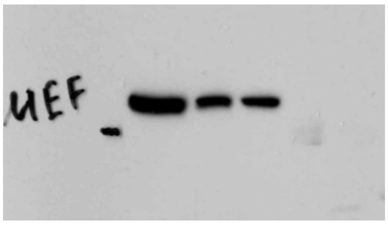

Figure 5.
A top segment of membrane was used for Plectin-1 blotting; pMEF cells.

Ch C P cN clN

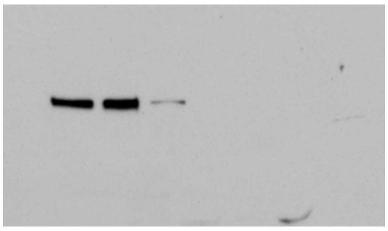

Note for Plectin-1 detection

Plectin is a giant protein (approx 500 kDa) found in all mammalian cells and acts as a link between the three main components of the cytoskeleton: actin, microtubules and intermediate filaments. Plectin was observed as predominantly cytosolic protein, however, a smaller part of the protein was fractionated with the perinuclear fraction.

Figure 2.
A medium segment of membrane was used for Nesprin-3 blotting; MDA-MB-435 cells.

Ch C P cN clN

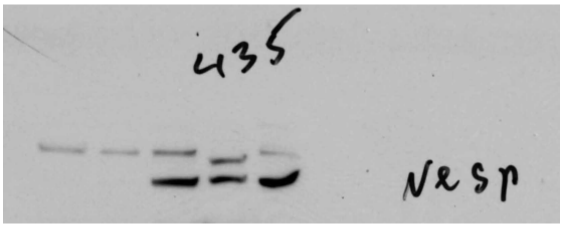

Figure 3.
A medium segment of membrane was used for Nesprin-3 blotting; HeLa cells.

Ch C P cN clN

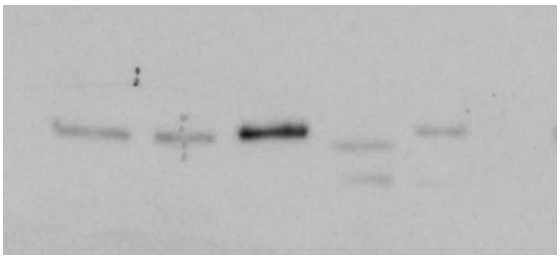

Figure 4.
A medium segment of membrane was used for Nesprin-3 blotting; MEF cells.

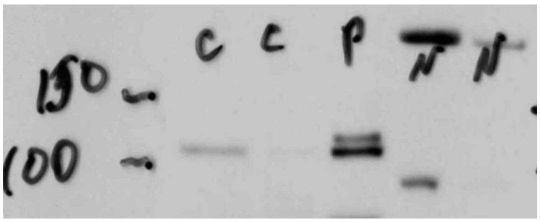

Figure 5.
A medium segment of membrane was used for Nesprin-3 blotting; pMEF cells.

Ch C P cN clN

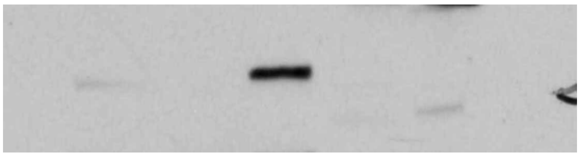

Note for Nesprin-3 detection

Nesprin-3 binds to the plakin family member plectin, which can associate with the intermediate filament (IF) system. Nesprin-3 was detected predominantly in perinuclear fraction.

Figure 2.
A medium segment of membrane was used for Sun2 blotting; MDA-MB-435 cells.

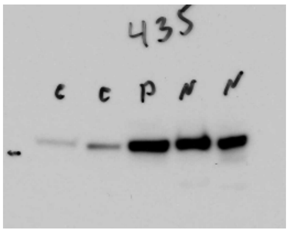

Figure 3.
A medium segment of membrane was used for Sun2 blotting; HeLa cells.

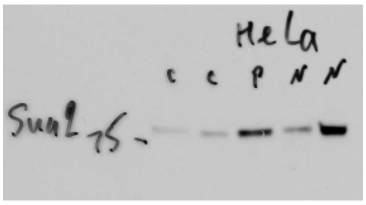

Figure 4.
A medium segment of membrane was used for Sun2 blotting; MEF cells.

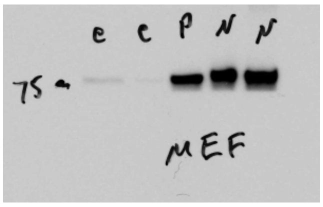

Figure 5.
A medium segment of membrane was used for Sun2 blotting; pMEF cells.

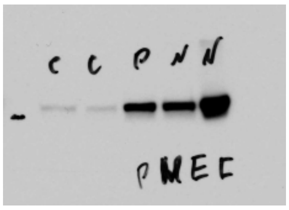

Note for Sun2 detection

Sun2 is a mammalian inner nuclear membrane protein. CDS technique extracts Sun2 to PNF in HeLa cells and partially extracts the protein to the perinuclear fraction in MEF and MDA-MB-435 cells.

For suggestions of Reviewer 2:

Figure 2.
A medium segment of membrane was used for PKCα blotting; MDA-MB-435 cells.

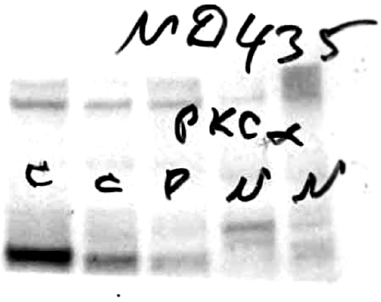

Figure 3.
A medium segment of membrane was used for PKCα blotting; HeLa cells.

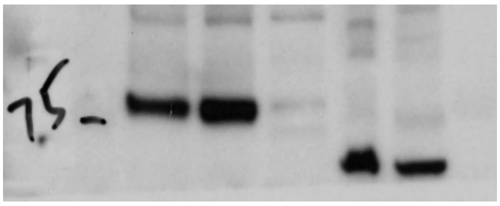

Figure 4.
A medium segment of membrane was used for PKCα blotting; MEF cells.

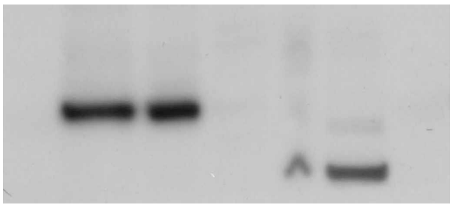

Figure 5.
A medium segment of membrane was used for PKCα blotting; pMEF cells.

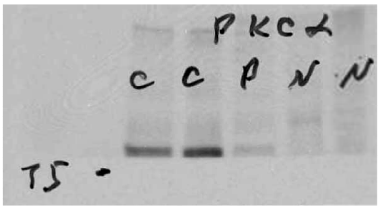

Note for PKCα detection:

Protein kinase C-alpha (PKC-α) is a specific member of serine- and threonine-specific protein kinase family. Under normal non-stimulated growth condition PKC-α is localized in the cytosol fraction.

Figure 2.
A medium segment of membrane was used for STAT3 blotting; MDA-MB-435 cells.

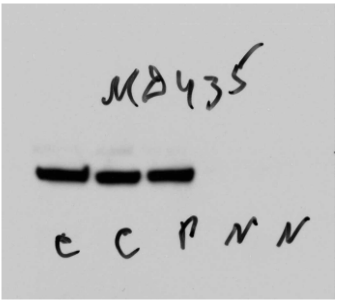

Figure 3.
A medium segment of membrane was used for STAT3 blotting; HeLa cells.

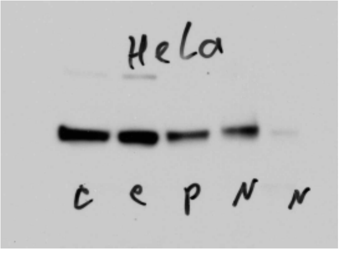

Figure 4.
A medium segment of membrane was used for STAT3 blotting; MEF cells.

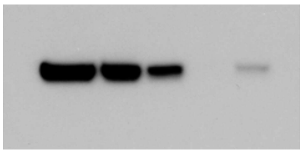

Figure 5.
A medium segment of membrane was used for STAT3 blotting; pMEF cells.

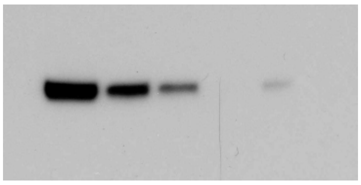

Note for STAT3 detection:

With the exception of HeLa cells in normal non-stimulated condition STAT3 localized in cytosol and perinuclear fraction of the cell. In HeLa cells it also localized in nucleus under non-stimulated condition.

Figure 2.
A bottom segment of membrane was used for ERK 42/44 blotting; MDA-MB-435 cells.

Ch C P cN clN

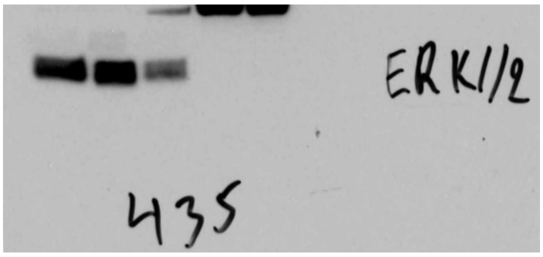

Figure 3.
A bottom segment of membrane was used for ERK 42/44 blotting; HeLa cells.

Ch C P cN clN

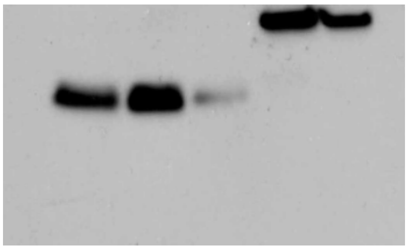

Figure 4.
A bottom segment of membrane was used for ERK 42/44 blotting; MEF cells.

Ch C P cN clN

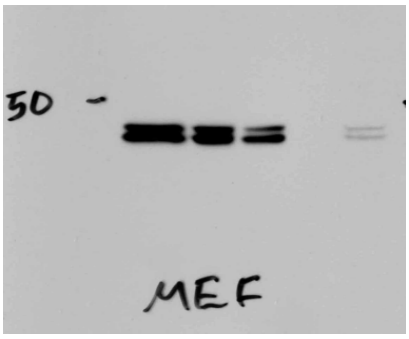

Figure 5.
A bottom segment of membrane was used for ERK 42/44b lotting; pMEF cells.

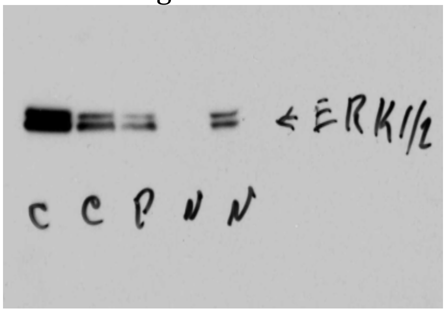

Note for ERK 42/44 detection:
The p44/42 MAPK (ERK1/2) signaling pathway can be activated in response to a diverse range of extracellular stimuli including mitogens, growth factors, and cytokines. In normal non-stimulated ERK 42/44 was detected in cytosol and perinuclear fractions, with the exception of HeLa cells where it was detected predominantly in the cytosol.

Figure 2.
A bottom segment of membrane was used for BCL10 blotting; MDA-MB-435 cells.

Ch C P cN clN

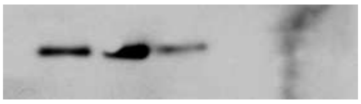

Figure 3.
A bottom segment of membrane was used for BCL10 blotting; HeLa cells.

Ch C P cN clN

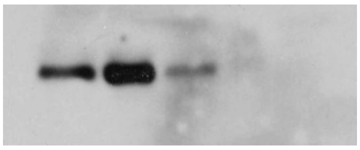

Figure 4.
A bottom segment of membrane was used for BCL10 blotting; MEF cells.

Ch C P cN clN

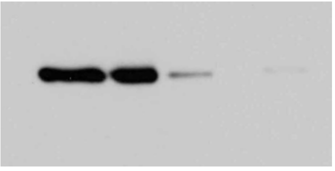

Figure 5.
A bottom segment of membrane was used for BCL10 blotting; pMEF cells.

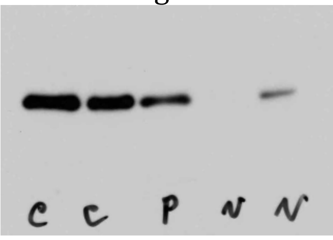

Note for BCL10 detection:
B-cell lymphoma/leukemia 10 contains a caspase recruitment domain (CARD), and has been shown to induce apoptosis and to activate NF-kappa B. With the exception of primary MEF cells BCL10 protein was detected in cytosol, however in pMEF cells the protein was detected also in the perinucleus.

Figure 2.
A medium segment of membrane was used for PLD1 blotting; MDA-MB-435 cells.

Ch C P cN clN

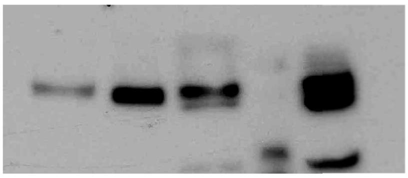

Figure 3.
A medium segment of membrane was used for PLD1 blotting; HeLa cells.

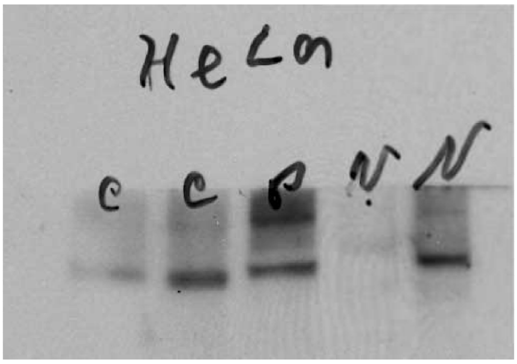

Figure 4.
A medium segment of membrane was used for PLD1 blotting; MEF cell

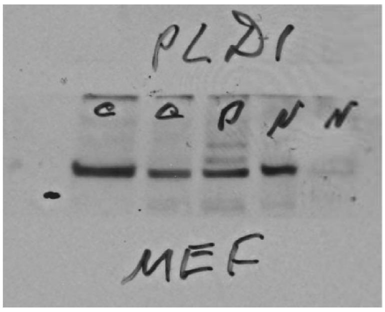

Figure 5.
A medium segment of membrane was used for PLD1 blotting; pMEF cells.

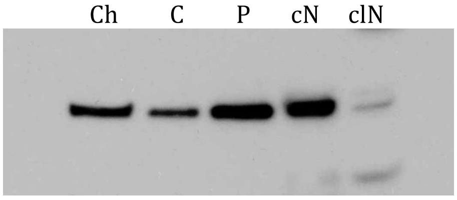

Note for PLD1 detection:

PLD1 is phosphatidylcholine-specific phospholipase, which catalyzes the hydrolysis of phosphatidylcholine in order to yield phosphatidic acid and choline. The enzyme may play a role in signal transduction and subcellular trafficking. PLD1 was detected in all cellular fractions of MEF cells, in primary cells was more concentrated in PNF but in immortalized MEF cells it shows also slow migratory bands in PNF. In MDA-MB-435 and HeLa cells it appeared in cytosole as well as in PNF.
